# Diet Quality: A Neglected Parameter in Children With Food Allergies. A Cross–Sectional Study

**DOI:** 10.3389/fped.2021.658778

**Published:** 2021-04-23

**Authors:** Aliki Kalmpourtzidou, Ioannis Xinias, Charalampos Agakidis, Antigoni Mavroudi, Dimitrios Mouselimis, Anastasios Tsarouchas, Eleni Agakidou, Thomai Karagiozoglou-Lampoudi

**Affiliations:** ^1^Department of Nutritional Sciences and Dietetics, School of Health Sciences, International Hellenic University, Thermi, Greece; ^2^3rd Department of Pediatrics, Faculty of Medicine, Aristotle University of Thessaloniki, Ippokrateion General Hospital, Thessaloniki, Greece; ^3^1st Department of Pediatrics, Faculty of Medicine, Aristotle University of Thessaloniki, Ippokrateion General Hospital, Thessaloniki, Greece; ^4^1st Department of Neonatology & Neonatal Intensive Care Unit (NICU), Faculty of Medicine, Aristotle University of Thessaloniki, Ippokrateion General Hospital, Thessaloniki, Greece

**Keywords:** children, diet quality, DQI-I, elimination diets, food allergy, macronutrient intake, micronutrient intake

## Abstract

**Background-Objective:** With recent evidence suggesting that growth is no longer considered a major issue in children with food allergies (FA) on elimination diet, priority has shifted to diet quality to establish healthy eating patterns and prevent non-communicable diseases. The Diet Quality Index – International (DQI-I) could be useful for assessing the overall diet quality of FA-children. This study aimed to evaluate the impact of elimination diet on DQI-I in children with FA and the accuracy of DQI-I in reflecting nutrient intake.

**Materials-methods:** In a prospective, cross-sectional, cohort study of FA-children (2–14 years), nutritional intake was evaluated using a 7-day food frequency questionnaire, 24-h dietary recall, and the DQI-I.

**Results:** Of the 76 children recruited, 44.7% had multiple allergies. Mean overall DQI-I score was 52 points, with only 28% of participants having good overall DQI-I (≥60 points). DQI-I moderation and balance were the most affected domains. Participants with multiple allergies had higher DQI-I moderation and balance and lower vitamin D and Ca intake. Compared to toddlers, schoolchildren had higher DQI-I variety and lower moderation and received higher vitamin B2, vitamin B12, Ca, P, and Zn. The number of allergies, age, and milk avoidance were independently associated with adjusted DQI-I moderation and balance, energy, and certain micronutrient intake. Higher percentages of participants with good DQI-I received adequate amounts of Mn and vitamins A, B6, C, and folate than those with poor DQI-I.

**Conclusions:** In children with FA on elimination diet, the DQI-I accurately captured the deflection of diet quality related to the development of chronic, non-communicable diseases through its moderation and balance components. This is DQI-I's main purpose as a healthy diet indicator and as such it would be a useful tool responding to the needs of the contemporary shifting of priorities in FA-children's diet from quantity to quality. Nevertheless, it does not accurately reflect the intake of certain micronutrients potentially compromised by elimination diets. Therefore, regular nutritional assessment utilizing both the DQI-I and tools assessing individual nutrient intakes along with professional nutrition counseling should be integral parts of the individualized management of children with FA to ensure adequate nutrient intake and establish healthy dietary patterns.

## Introduction

Elimination diets are central in the management of food allergies (FA) (1, 2). However, avoidance of specific foods may be a difficult task for children, while the high rate of growth renders them susceptible to energy, macronutrient, and micronutrient insufficiency (3, 4). Previous studies in children with FA showed that elimination diet could compromise the intake of energy, macronutrients, especially proteins, and essential fatty acids, as well as specific micronutrients intake, mainly Ca and vitamin D, while low intake of vitamin E, and iodine have also been observed (5–11). Kim et al. studied a cohort of patients (age range 1 to 65 years) with atopic dermatitis on elimination diet (12). It was found that compared to patients with negative oral food challenge test those with positive test results received lower amounts of vitamins (A, B1, B2, B3, B6, and K), minerals (Ca, P, K), and trace elements (Fe, Zn), depending on the kind of the avoided foods (12). In addition, quality of life was compromised in children on elimination diet, especially when dietary advise was not received (13). With recent evidence suggesting that growth issues are no longer considered as a major problem in pediatric patients with FA on elimination diet, the significance of establishing healthy eating patterns to prevent non-communicable diseases makes the diet quality during this sensitive period of life ever so important, shifting priority from quantity to quality (4, 14–16). This priority highlights the importance of accurately assessing the diet quality of children with FA utilizing tools appropriate for pediatric population (17).

The Diet Quality Index – International (DQI-I) can be calculated using a variety of dietary assessment methods and was developed for global monitoring of diet quality across countries (18). Currently, limited studies utilized the DQI-I for evaluation of diet quality in healthy children and adolescents (19–22), as well as children with certain pathologic conditions, such as cerebral palsy, liver transplantation (23, 24). Williams et al. who assessed the DQI-I in a cohort of adolescents (12–18 years old) attending secondary school found that the DQI-I total score was 53.7 and 51.3 in boys and girls, respectively (19). Silva et al. in a cohort of healthy children and adolescents aged 10–17 years showed that the DQI-I score (mean 49.6 points) was positively correlated with physical well-being (20). A survey in Nova Scotia by Colapinto et al. examining the lifestyle of 4,966 children associated the consumption of large quantities of French fries and potato chips with lower diet quality assessed using the DQI-I (21). In a group of healthy children aged 9–10 years, with 33% of them being overweight/obese, Gaskin et al. found that the mean DQI-I score was 57 points, while only 37% of the study population attained a score of 60% or higher (22). Karagiozoglou–Lampoudi et al. in a survey of children with cerebral palsy found that 62% of them had an average DQI-I with 28% having a good DQI-I (equal to or higher than 60 points), while the DQI-I was significantly correlated with energy intake to requirement ratio and macronutrient intake (23). Alzaben et al. reported that the DQI-I in a group of children who underwent liver transplantation was comparable to that of healthy controls (24). However, data on the effect of FA and elimination diet on DQI-I and DQI-I domains of children are lacking. The primary aim of the present study was to assess the diet quality using the DQI-I in children with FA and the ability of the DQI-I to accurately reflect the adequacy of specific nutrient intakes. The secondary aim was to evaluate the potential effect of the number of allergies, age, and the kind of foods that are avoided on DQI-I and nutrient intake. To this aim, the DQI-I, energy, macronutrient, and micronutrient intakes were assessed in a cohort of children with FA on elimination diet.

## Methods and Study Population

### Study Design and Population

This is an observational, prospective, cross-sectional cohort study of 76 children aged 2–14 years with FA previously diagnosed, who were treated with elimination diet, hereafter referred to as participants. Participants were recruited during their visits to the pediatric allergy and gastroenterology outpatient clinics of two tertiary pediatric departments from December 2018 to November 2019. Criteria for diagnosis of FA included medical history and clinical findings compatible with FA, food-specific IgE (RAST), skin prick test, and the response to elimination diet, while it was documented by a positive oral food challenge in selected cases, according to the guidelines prepared by the European Academy of Allergy and Clinical Immunology's (EAACI) Guidelines for Food Allergy and Anaphylaxis Group (25). Exclusion criteria were co-morbidities that could affect the patients' quality of diet independently of the presence of FA, including gastrointestinal and other chronic diseases (e.g., cerebral palsy, congenital heart disease, diabetes, chronic kidney disease) and genetic syndromes, as well as the refusal of parental consent. Before recruitment, the patient's records were reviewed for completeness and to examine whether the tests performed for diagnosis of FA fulfilled the criteria set for inclusion in the current study. Detailed medical history and information regarding previous nutritional counseling and tests supporting the diagnosis of FA were obtained from the patients or their parents and the medical records. Physical examination, anthropometry, and dietary evaluation were performed at the time of their visit to the outpatient clinic. The cohort was divided into subgroups according to: (a) the DQI-I score, into poor (DQI-I score <60 points) and good DQI-I (DQI-I score =/>60 points) subgroups; (b) the number of FA, into single-allergy and the multiple-allergy (two or more FA) subgroups; (c) the age, into toddlers (aged 2–4 years) and schoolchildren (aged 5–14 years). The study was approved by the Ethical Committee of our Institution and informed consent was obtained from all parents.

### Methods

The medical history related to FA was recorded by either a pediatric gastroenterologist or a pediatric allergist who also performed clinical examination and anthropometric measurements. The height and weight were measured using standard procedures and measurements were converted to age- and sex- specific z-scores of weight (weight for age z score) and height (height for age z score) using the software WHO Anthro v.3.2.2 for children 0–5 years old and WHO Anthro Plus v.1.0.4 for children 5–14 years old.

#### Dietary Assessment

A 5-pass 24-h recall and a validated semi-quantitative FFQ translated in Greek language were used for the dietary assessment of children with FA. The FFQ consists of a total of 68 questions; 10 questions about dairy products, five about fruits, 11 about vegetables, 10 about animal deriving food sources (meat, fish, eggs), nine about bakery products, cereals and deserts, 23 about other food products like alcohol, coffee, tea, sugar, chips etc (26). Energy and nutrient intake were calculated by a Dietary Analysis software Food Processor v7.30 (ESHA, Portland, OR). The age – and sex- specific energy requirements were calculated using WHO equations (27). The daily energy intake was expressed as percentage of the recommended age-specific daily requirements (IR%). We calculated the percentage of daily energy intake (E%) derived from carbohydrates, proteins, and fat, and the ratio of unsaturated to saturated fatty acid intake defined as intake of mono-unsaturated fatty acid (MUFA) plus poly-unsaturated fatty acid intake (PUFA) divided by the saturated fatty acid (SFA) intake ([PUFA + MUFA]/SFA). In addition, the percentage of participants with a ratio of macronutrient-derived E% (carbohydrate:protein:fat) within the DQI-I recommended range was calculated.

The energy, macronutrient, and micronutrient intake data were analyzed against the Dietary Reference Values for the European Union population published by the European Food Safety Authority (EFSA) Panel on Dietetic Products, Nutrition and Allergies (NDA) (28). The recommended values of either the adequate intake (AI), when available, or alternatively, the average requirements (AR) were used as cutoff points for assessing the adequacy of micronutrient intake. For total energy IR% and carbohydrate- and fat–derived energy the reference ranges were available. According to the EFSA–NDA panel recommendations, the AI represents “the average daily level of nutrient intake by a reference population of apparently healthy people that is assumed to be adequate,” while the AR represents “the level of intake that is adequate for half of the people in a reference population group” (28). Values are presented as percentage of participants with adequate intake, which was defined as intake either within the reference range or above the lower reference intakes, depending on the way the published reference values are expressed.

#### Diet Quality Assessment

The DQI-I was used for the assessment of diet quality. A detailed description of the DQI-I has been published previously by Kim et al. (18). In short, this tool comprises 17 items grouped into four domains covering the aspects of healthy diet variety, adequacy, moderation, and overall balance. The overall DQI-I score ranges from 0 (lowest) to 100 points (perfect). Good diet quality has been defined as an overall DQI-I score of 60 points or higher (18, 20). Variety contains two items assessing the diversity of nutrient sources both across and within food groups. The variety score ranges from 0 (worst) to 20 points (best). Adequacy includes eight items evaluating the adequacy of intake of certain food groups and nutrients that must be supplied in sufficient amounts to ensure a healthy diet and prevent malnutrition. Adequacy score ranges from 0 (worst) to 40 (best). Moderation contains five items that evaluate the intake of food and nutrients which are associated with the occurrence of chronic diseases, such as total and saturated fat, cholesterol, salt, and empty foods. As the excessive intake of these nutrients increases the risk of chronic diseases, the highest intake of each item in the moderation domain is given the lowest score (0 points) while the lowest intake is given the highest score (6 points) resulting in a total moderation score range from 0 (worst) to 30 points (best). The DQI-I overall balance domain examines the balance among energy sources (carbohydrates:proteins:fat) and fatty acids (PUFA:MUFA:SFA) comprising two items given a maximum of 10 points (18, 20).

### Statistical Analysis

Values are presented as means and standard deviations or medians and interquartile ranges (IQR), depending on value distribution. Although the distribution of DQI-I variety, moderation, and balance values was non-Gaussian, the overall DQI-I and domains are presented as mean (±SD) to facilitate comparisons with the mean values reported by previous authors (20, 29, 30). Categorical variables are presented as counts and percentages. Comparisons between groups were performed using the Mann-Whitney U test and the Fisher's exact test, as appropriate. The Spearman Correlation Coefficient was used to correlate the DQI-I score with the micronutrient intake. Separate multiple regression analysis models were constructed for the DQI-I and DQI-I domain scores, as well as for energy and each macronutrient and micronutrient intake. The number of allergies, age, and sex entered all models as independent variables. Due to the significant bivariate differences between participants on free-milk diet and those with other food allergies, all regression models were tested twice, i.e., with and without milk avoidance as a cofactor, in order to clarify the potential effect of milk avoidance on the associations of the number of allergies and age with diet quality and nutrient intake. Multiple regression analysis was performed using Generalized Linear Models with logarithmic transformation of variables with non-Gaussian distribution. The level of significance was set at *p*-value < 0.05. Statistical analysis was performed using the software IBM SPSS v. 23.

#### Sample Size and *post-hoc* Power

The rationale for using this certain cohort of 76 participants was largely practical: It was determined by the number of children with previously diagnosed FA that visited the hospital gastroenterology and allergy clinics during the study period and met the inclusion criteria. *Post-hoc* analysis was performed using the online calculator ClinCalc (available at https://clincalc.com/stats/samplesize.aspx, accessed on 12/03/2021). *Post-hoc* power analysis was performed to assess the power of the study to detect significant differences concerning the primary outcome measures, namely the DQI-I and DQI-I domains, between the compared subgroups. Analysis showed that the power of the study to detect significant differences between the subgroups with poor/good DQI-I was high as for the overall DQI-I, variety, and moderation domain scores (100, 90, and 100%, respectively), and moderate as for the DQI-I overall balance (76%). In addition, the study power to discriminate differences in DQI moderation between the subgroups with milk allergy and those with other FA was high (99%). The power for detecting other significant differences in overall DQI-I and domains between the compared subgroups was low (Please see Supplementary Table 1).

## Results

### Epidemiological Data

The median age at assessment was 5 years (range 2–14 years) with 46% being toddlers (2–4 years) and 54% schoolchildren (Table 1). Of the participants, 42 (55.3%) had a single FA, 21 (27.6%) two FA, and 13 (17.1%) three or more food allergies. Cow's milk protein (52.6%), egg (47.4%), nuts (23.7%), and fish (14.5%) were the most common foods responsible for allergic reactions in this study population. Gastrointestinal symptoms were observed in 78% of the participants, followed by dermatological manifestations in 57% of them. All the participants' parents had received standard, written nutritional instructions by the attending allergist/gastroenterologist. There was no significant difference between the subgroups with single or multiple allergies as to the age and sex distribution (Table 1). The mean z-scores of weight and height of the cohort were within the normal range. In line with this finding, only 5.3% of participants were underweight (weight for age z score < −2SDs), 5.3% were stunted (height for age z score < −2SDs), while 25% were overweight (weight for age z score > +2SDs), without any significant difference between the single- and multiple-allergy subgroups.

**Table 1:** Demographics and scores of DQI-I and DQI-I domains of the total cohort and the subgroups related to DQI-I categories, number of allergies, and age.

	**Cohort**	**Poor DQI-I**	**Good DQI-I**	***p***	**Single allergy**	**Multiple allergies**	***p***	**Toddlers**	**School children**	***p***
N	76	55	21		42	34		35	41	
Multiple allergies (*n*, %)	34 (44.8)	22 (64.7)	12 (53.3)	0.205	0 (0)	34 (100)	N.A.	18 (51.4)	16 (39.0)	0.278
Age (years)^*^	5.0 (4.5)	5.0 (5.0)	4.5 (3.0)	0.456	5.0 (5.0)	4.0 (3.8)	0.468	3.0 (0.5)	7.2 (3.4)	**<0.001**
Male sex (*n*, %)	28 (36.8)	20 (36.6)	8 (38.1)	1.0	17 (40.5)	11 (32.4)	0.485	11 (31.4)	17 (41.5)	0.475
Female sex (*n*, %)	48 (432.2)	35 (63.6)	13 (61.9)		25 (59.5)	23 (67.6)		24 (68.6)	24 (58.5)	
Overall DQI-I points^#^	52.1 ± 10.0	47.4 ± 7.0	64.6 ± 4.0	**<0.001**	51.0 ± 8.7	53.5 ± 11.3	0.230	52.7 ± 10.4	51.7 ± 9.7	0.539
DQI-I variety points^#^	12.3 ± 4.8	11.3 ± 4.8	14.8 ± 3.7	**0.003**	12.3 ± 5.1	12.3 ± 4.5	0.836	11.2 ± 4.2	13.2 ± 5.1	**0.045**
DQI-I adequacy points^#^	24.0 ± 6.1	22.7 ± 6.1	27.5 ± 4.6	**0.001**	24.7 ± 6.1	23.2 ± 6.0	0.350	24.9 ± 5.8	23.2 ± 6.3	0.243
DQI-I moderation points^#^	13.8 ± 4.9	13.4 ± 3.9	18.7 ± 5.3	**<0.001**	13.6 ± 4.6	16.3 ± 5.1	**0.014**	16.1 ± 5.1	13.8 ± 4.5	**0.041**
DQI-I balance points^#^	0.87 ± 1.7	0.51 ± 1.5	1.8 ± 2.0	**<0.001**	0.71 ± 1.8	1.1 ± 1.6	**0.047**	1.0 ± 1.8	0.73 ± 1.7	0.294

# *, mean± SD;*

* *, median (interquartile range); N.A., not applicable. The statistically significant differences are shown in bold letters*.

### Overall DQI-I and Domain Points of the Total Cohort

**Figure 1:**
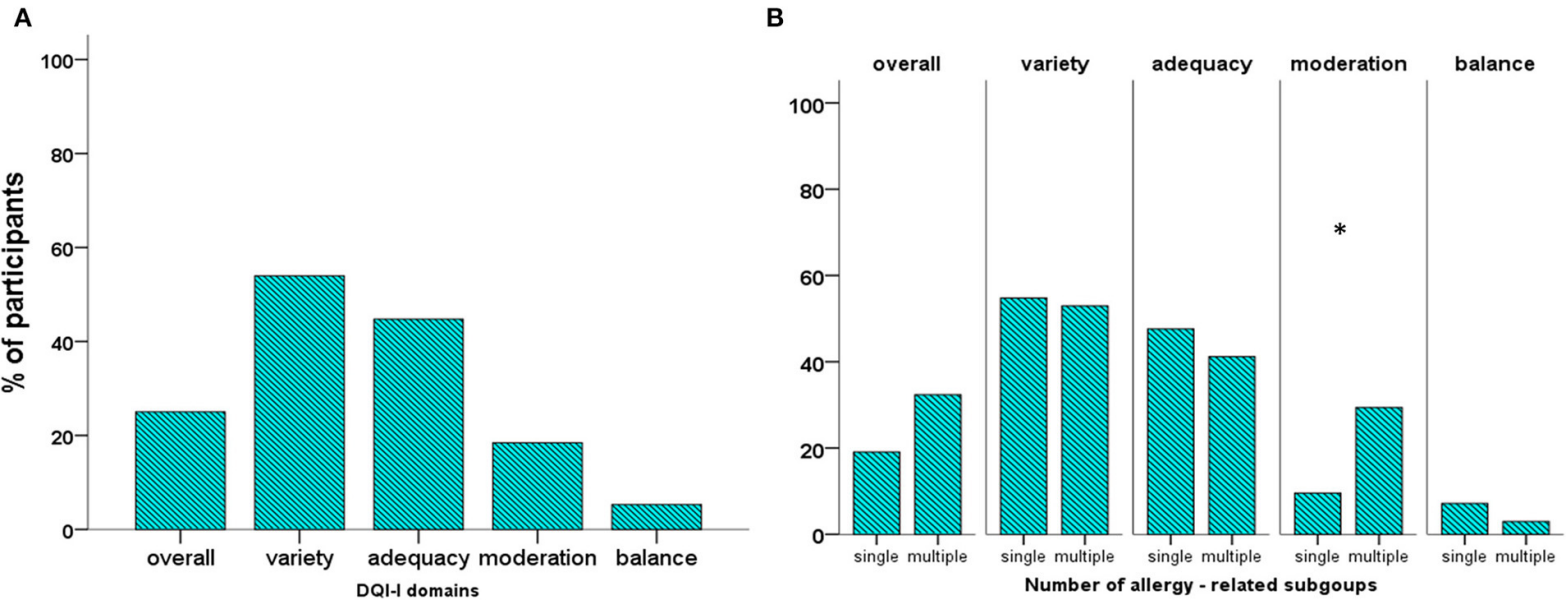
Percentage of participants with overall DQI-I and DQI-I domain score higher than or equal to 60% of the perfect score in the cohort **(A)** and the subgroups related to the number of allergies **(B)**. *p < 0.05.

The mean overall DQI-I score (52 points) was marginally lower than the cutoff score of good quality of diet (60 points) (Table 1, Supplementary Figure 1). Only 28% of the study population had good overall DQI-I (Table 1, Figure 1A). Of the four DQI-I domains, mean scores of adequacy, moderation, and overall balance were lower than the respective cutoff points of good quality (<60% of the respective perfect score), while the variety score was marginally higher than this level (Table 1). The percentage of participants with good DQI-I variety, adequacy, moderation, and balance was 53.9, 44.7, 18.4, and 5.3%, respectively (Figure 1A).

### Comparisons of DQI-I Between Subgroups

The mean overall DQI-I score and the proportions of participants with good overall diet quality did not differ significantly between the subgroups related to the number of allergies, age, and sex (Table 1 and Supplementary Table 2, Figure 1B and Supplementary Figure 2). Regarding the four domains of the DQI-I, the scores of moderation and balance were significantly higher in the multiple-allergy than in the single-allergy subgroup (*p* = 0.014 and *p* = 0.047, respectively, Table 1). Moreover, a higher proportion the multiple-allergy subgroup had good DQI-I moderation, compared to the single-allergy one (29.4 vs. 9.5%, *p* = 0.037, Figure 1B). Toddlers had significantly lower variety and higher moderation scores than schoolchildren (*p* = 0.045 and 0.041, respectively), while comparable proportions of toddlers and schoolchildren had good DQI-I domain scores (Table 1, Supplementary Figure 2). Neither the overall DQI-I nor the DQI-I domain scores differed significantly between males and females (Supplementary Table 2).

### Energy and Macronutrient Intake

**Figure 2:**
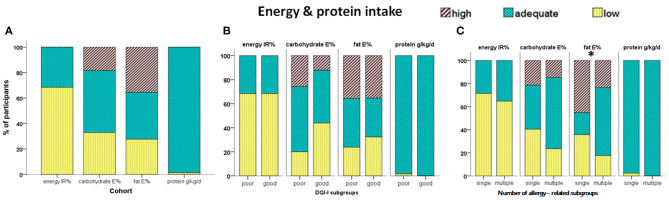
Percentage of participants receiving adequate, excess, or low total energy (IR%, intake as percentage of requirements), carbohydrate- and fat-derived E% (energy as percentage of total daily energy intake), and protein (g/kg/d) in the cohort **(A)**, the subgroups with poor / good overall quality of diet (good = overall DQI-I =/> 60 points) **(B)** and the subgroups related to the number of allergies **(C)**. *p < 0.05.

The data on energy and macronutrient intake are summarized in Table 2 and Supplementary Table 2 and are depicted in Figures 2A–C and Supplementary Figure 3. The recommended daily energy (IR%) and protein intake were achieved by 32 and 98.7% of the participants, respectively. The recommended carbohydrate E% was met by 48,7% of the cohort, while 32.9 and 18.4% received lower and higher, respectively carbohydrate. The recommended fat E% was met by 36.8% of the participants, while 27.6 and 35.5% received lower and higher, respectively fat E% (Figure 2A). Participants with good DQI-I received significantly higher daily carbohydrate amounts per kilogram of body weight and lower saturated fatty acid (SFA) E% (Table 2). The proportions of participants receiving adequate IR% and protein as well as those receiving carbohydrate E% and fat E% within the reference range did not differ significantly between the good and poor DQI-I subgroups (Figure 2B).

**Table 2:** Energy and macronutrient daily intake by the total cohort and the subgroups related to DQI-I categories, number of allergies, and age.

	**Cohort**	**Poor DQI-I**	**Good DQI-I**	***p***	**Single allergy**	**Multiple allergies**	***P***	**Toddler**	**School children**	***P***
Carbohydrate (g/kg/d)^*^	8.3 (7.0)	6.8 (7.6)	9.7 (5.0)	**0.017**	8.1 (7.6)	8.3 (7.0)	0.683	11.3 (5.8)	5.3 (5.1)	**<0.001**
Protein (g/kg/d)^*^	2.4 (1.4)	2.3 (1.6)	2.6 (1.4)	0.203	2.4 (1.5)	2.3 (1.3)	0.920	2.8 (2.3)	2.3 (4.9)	**0.012**
Fat (g/kg/d)^*^	2.1 (1.3)	2.1 (1.5)	2.3 (1.7)	0.243	2.0 (1.6)	2.2 (0.9)	0.381	2.3 (1.7)	2.0 (1.5)	**0.020**
Energy IR %^#^	86.7 ± 30.5	82.9 ± 30.9	96.6 ± 25.7	0.081	84.0 ± 30.1	90.5 ± 30.4	0.543	98.6 ± 28.5	76.6 ± 28.1	**0.001**
Carbohydrate E%^#^	50.1 ± 13.1	48.9 ± 13.9	53.2 ± 10.2	0.122	50.2 ± 14.6	49.9 ± 11.0	0.829	54.7 ± 13.6	46.3 ± 11.7	**0.003**
Protein E%^#^	17.2 ± 6.1	17.8 ± 6.6	15.8 ± 4.6	0.292	17.5 ± 5.7	16.9 ± 6.6	0.440	15.7 ± 6.7	18.3 ± 5.2	**0.007**
Fat E%^#^	32.7 ± 9.7	33.3 ± 10.2	31.0 ± 8.3	0.198	32.3 ± 11.2	33.2 ± 7.7	0.986	29.7 ± 9.7	35.4 ± 9.4	**0.005**
SFA E%^#^	12.2 ± 6.40.2	13.1 ± 6.3	9.7 ± 6.2	**0.026**	13.0 ± 5.5	11.2 ± 6.2	0.209	9.6 (8.7)	14.3 (10.9)	**0.012**
SFA (g/d)^*^	14.6 (14.5)	15.3 (16.3)	10.1 (11.5)	0.104	15.4 (14.5)	13.3 (8.5)	0.233	11.9 (1.0)	18.5 (16.2)	**0.001**
MUFA (g/d)^*^	12.9 (12.2)	12.7 (12.5)	13.8 (10.5)	0.610	13.5 (18.9)	12.2 (11.9)	0.886	12.7 (8.9)	16.0 (12.0)	0.059
PUFA (g/d)^*^	4.5 (4.5)	4.6 (4.6)	4.2 (4.3)	0.924	4.4 (4.1)	5.4 (4.8)	0.538	4.0 (4.6)	5.0 (4.0)	0.077
MUFA:PUFA:SFA^*^	1.2 (1.1)	(0.9)	1.8 (2.1)	0.083	1.1 (0.9)	1.5 (1.4)	0.357	1.3 (1.5)	1.1 (1.0)	0.334
Omega 3 (g/d)^*^	0.33 (0.26)	0.34 (0.26)	0.30 (0.30)	0.833	0.36 (0.31)	0.30 (0.27)	0.094	0.27 (0.24)	0.38 (0.34)	**0.002**
Omega 6 (g/d)^*^	2.75 (3.4)	2.6 (3.4)	3.3 (3.3)	0.271	2.6 (3.1)	2.9 (4.07)	0.602	2.4 (2.7)	3.3 (3.3)	0.143
Trans (g/d)^*^	0.46 (0.8)	0.45 (0.73)	0.54 (0.99)	0.490	0.46 (0.8)	0.46 (0.7)	0.874	0.46 (0.64)	0.47 (1.1)	0.509
Cholesterol (mg/d)^*^	120 (140)	123 (159)	113 (84)	0.107	137 (144)	94.9 (128)	0.122	81 (141)	145 (129)	**0.012**

# *, mean± SD;*

* *, median (interquartile range); E%, energy as percentage of total energy intake; IR%, intake as percentage of requirements; MUFA, Mono-Unsaturated Fatty Acids; PUFA, Poly-Unsaturated Fatty Acids; SFA, Saturated Fatty Acids. The statistically significant differences are shown in bold letters*.

Analysis according to the number of allergies showed that energy and macronutrient intake did not differ significantly between the single-allergy and multiple-allergy subgroups (Table 2). However, a significantly higher proportion of the multiple-allergy subgroup received Fat E% within the reference range and lower proportion received high fat E% compared to the single-allergy subgroup (*p* = 0.002, Figure 2C). Further analysis in relation to the two age-related subgroups showed that toddlers received significantly higher daily amounts of macronutrients per kilogram of body weight, energy IR%, and carbohydrate E%, and lower protein E%, fat E%, saturated fat, and omega 3 PUFA compared to schoolchildren (Table 2). In addition, significantly higher proportions of toddlers received total energy IR% over the age-specific reference values (*p* = 0.003), and carbohydrate E% within the reference range (*p* = 0.006), while higher proportion of schoolchildren received high amount of fat E% (*p* < 0.001) (Supplementary Figure 3). No significant differences in energy and macronutrient intake were found between males and females (Supplementary Table 2).

### Micronutrient Intake

#### Vitamin Intake

**Figure 3:**
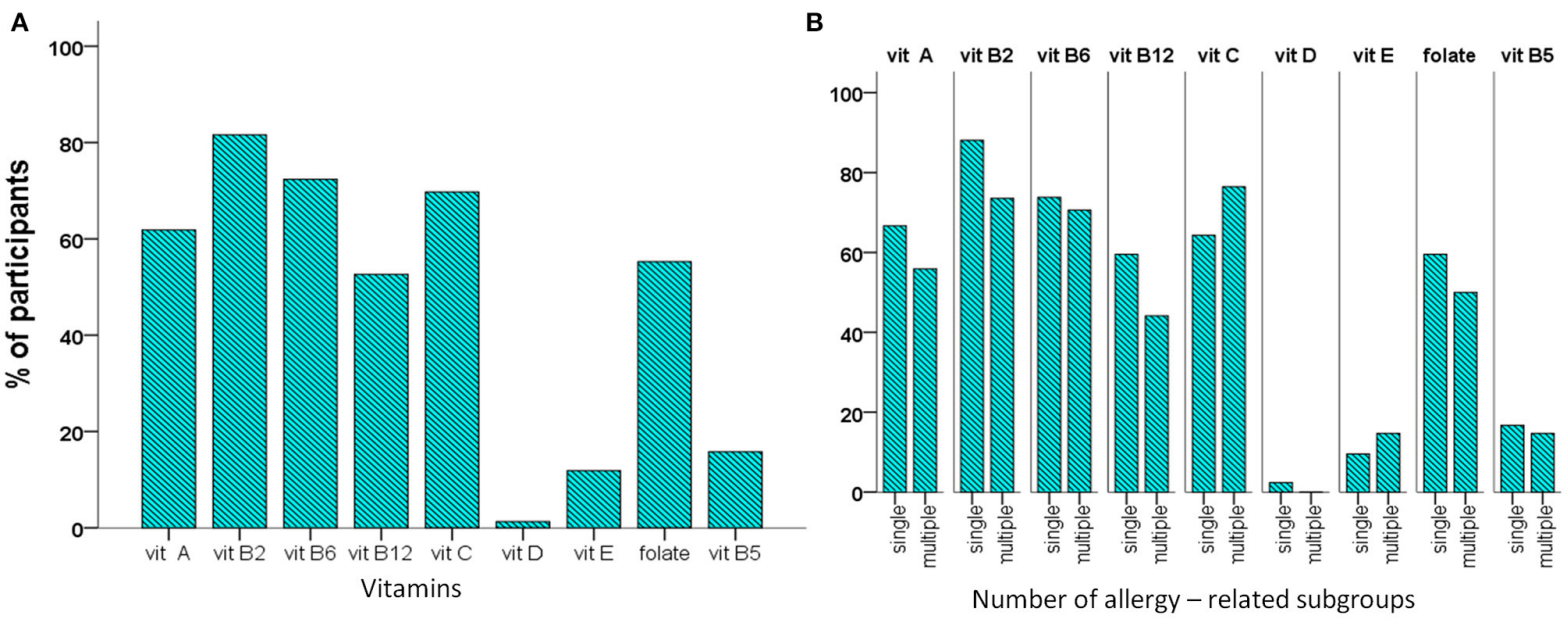
Percentage of participants with vitamin intake higher than the reference intake (RI) in the cohort **(A)**, and the subgroups related to the number of allergies **(B)**. There was no significant difference.

The daily amounts of vitamin intake are shown in Table 3 and the percentages of participants with daily vitamin intake higher than the reference AI/AR are depicted in Figure 3A. The percentage of participants with vitamin intake higher than the reference AI/AR levels of intake ranged between 100 (vitamin B1) and 1.3% (vitamin D). Comparisons between the subgroups with single and multiple allergies did not show any significant difference concerning either the median amount or the frequency of adequate vitamin intake (Table 3, Figure 3B). Compared to toddlers, the schoolchildren received significantly higher median amounts of the vitamins B2 (1.2 mg/d vs. 0.86 mg/d, *p* = 0.010) and B12 (2.3 μg/d vs. 1.2 μg/d, *p* = 0.001), while a lower proportion of them received the recommended intake of folate (39 vs. 74%, *p* = 0.003, Table 3, Supplementary Figure 4). Comparison between males and females showed that only folate intake differed significantly between sexes, being higher in males (*p* = 0.049, Supplementary Table 3).

**Table 3:** Micronutrient daily intake (median, interquartile range) by the total cohort and the subgroups related to DQI-I subcategories, number of allergies, and age.

**Median, IQR**	**Cohort**	**Poor DQI-I**	**Good DQI-I**	***p***	**Single allergy**	**Multiple allergies**	***P***	**Toddler**	**School children**	***P***
Vit. A (μg/d)	495 (752)	276 (569)	911 (941)	**<0.001**	541 (712)	398 (823)	0.345	521 (719)	356 (869)	0.762
Vit. B1 (mg/MJ)	0.81 (0.6)	0.77 (0.7)	0.85 (0.4)	0.732	0.80 (0.6)	0.82 (0.5)	0.965	0.70 (0.4)	0.90 (0.7)	0.121
Vit. B2 (mg/d)	1.14 (0.8)	1.17 (0.6)	0.85 (1.0)	0.251	1.2 (0.6)	0.86 (0.9)	0.061	0.86 (0.8)	1.2 (0.7)	**0.010**
Vit. B3 (mg/MJ)	9.3 (7.2)	9.1 (8.2)	11.5 (8.6)	0.064	8.8 (7.3)	10.2 (6.9)	0.130	8.8 (7.9)	9.6 (7.2)	0.146
Vit. B6 (mg/d)	0.86 (0.8)	0.81 (0.7)	1.2 (0.9)	0.110	0.8 (0.9)	0.9 (0.7)	0.961	0.80 (0.7)	0.95 (0.9)	0.204
Vit. B12 (μg/d)	2.0 (2.3)	2.1 (2.2)	1.6 (1.7)	0.137	2.1 (2.0)	1.7 (2.4)	0.160	1.2 (1.4)	2.3 (1.5)	**0.001**
Vit. C (mg/d)	40.1 (68)	38.4 (52)	48.5 (72)	0.097	47.1 (49)	38.0 (86)	0.856	41.1 (83)	38.7 (44)	0.494
Vit. D (μg/d)	2.9 (4.8)	3.1 (4.2)	0.9 (5.2)	0.128	3.3 (4.5)	1.0 (4.2)	**0.015**	1.5 (4.6)	3.1 (4.6)	0.233
Vit. E (mg/d)	2.5 (2.9)	2.2 (2.7)	3.1 (2.3)	0.054	2.7 (3.0)	2.3 (2.1)	0.292	2.8 (2.5)	2.3 (3.4)	0.696
Folate (μg/d)	117 (100)	130 (76)	153 (116)	0.055	126 (80)	111 (115)	0.457	130 (92)	113 (109)	0.764
Pantothenic acid (mg/d)	2.5 (1.4)	2.6 (1.3)	2.5 (1.5)	0.771	2.6 (1.3)	2.3 (1.4)	0.253	2.3 (1.1)	2.9 (1.6)	0.084
Ca (mg/d)	570 (547)	604 (432)	349 (726)	0.309	647 (492)	350 (620)	**0.016**	472 (557)	713 (521)	**0.002**
Cu (mg/d)	0.57 (0.4)	0.48 (0.5)	0.70 (0.4)	**0.001**	0.53 (0.4)	0.66 (0.5)	0.178	0.50 (0.5)	0.59 (0.4)	0.318
Fe (mg/d)	6.4 (3.9)	6.2 (3.4)	7.0 (4.1)	0.067	6.3 (4.2)	6.5 (3.7)	0.757	6.6 (4.0)	6.2 (3.9)	0.482
Mg (mg/d)	141 (74)	137 (77)	144 (69)	0.224	144 (67)	139 (92)	0.751	140 (84)	143 (78)	0.541
Mn (mg/d)	1.1 (1.0)	0.87 (0.8)	1.6 (0.9)	**0.001**	1.0 (1.0)	1.1 (1.0)	0.923	1.1 (1.4)	1.0 (0.8)	0.818
P (mg/d)	701 (423)	709 (409)	611 (518)	0.366	724 (387)	611 (552)	0.111	560 (396)	786 (368)	**0.009**
K (mg/d)	1529 (646)	1526 (577)	1632 (982)	0.556	1492 (550)	1529 (920)	0.530	1481 (583)	1539 (621)	0.180
Se (μg/d)	52 (58)	51.9 (58)	57.1 (57)	1.0	53 (57)	51 (59)	0.996	46 (62)	60 (53)	0.071
Zn (μg/d)	5.5 (4.2)	5.2 (4.3)	6.0 (3.7)	0.811	5.5 (3.6)	6.0 (4.4)	0.990	4.9 (3.6)	6.0 (4.2)	**0.033**
Na (mg/d)	920 (853)	916 (781)	959 (1044)	0.863	975 (975)	772 (702)	0.174	656 (597)	1164 (1053)	**0.001**

*The statistically significant differences are shown in bold letters*.

#### Mineral Intake

**Figure 4:**
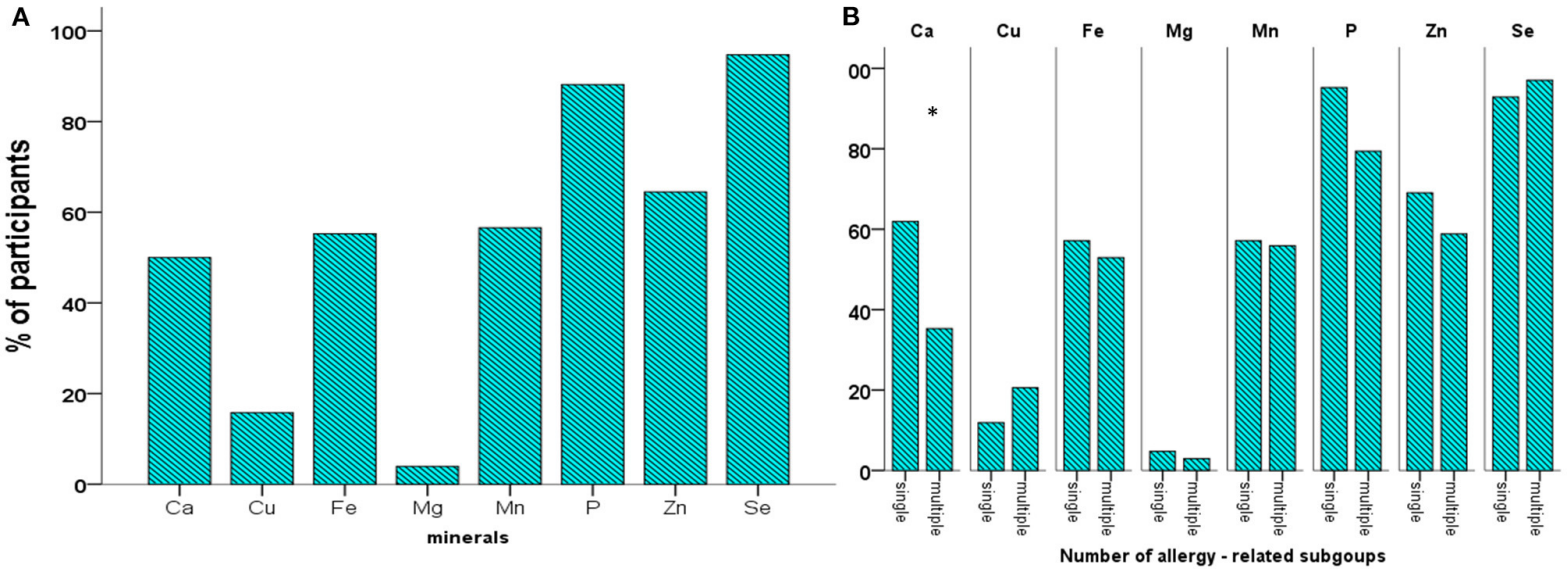
Percentage of participants with mineral intake higher than the reference intake (RI) in the cohort **(A)**, and the subgroups related to the number of allergies **(B)**. *p < 0.05).

The daily amounts of mineral intake are shown in Table 3, while the percentage of participants with daily mineral intake higher than the reference AI/AR is depicted in Figure 4A. The proportions of participants with mineral intake higher than the recommended AI /AR ranged between 3.9% (Mg) and 94.7% (Se) (Figure 4A). Comparisons between the single- and multiple-allergy subgroups showed that the multiple-allergy subgroup received lower median Ca intake (*p* = 0.016) while having adequate Ca intake less often (*p* = 0.037) (Table 3, Figure 4B). Further analysis in relation to age showed that schoolchildren received significantly higher daily amounts of Ca, P, Zn, and Na (*p* = 0.002, *p* = 0.009, *p* = 0.033, and *p* = 0.001, respectively, Table 3). On the other hand, a significantly lower percentage of schoolchildren had adequate Fe, Mn, and K intake than the toddlers (*p* = 0.011, *p* = 0.005, and *p* < 0.001, respectively, Supplementary Figure 5). There was no significant difference between males and females as to either the daily intake of minerals (Supplementary Table 3) or the proportion receiving the recommended intake (data not shown).

### Analysis in Relation to the Kind of Avoided Foods

Further analysis was performed in relation to the foods most commonly avoided by the participants, specifically the milk and egg. Comparisons between participants with milk allergy and those with non-milk FA showed significant differences in age, overall DQI-I and DQI-I moderation and balance scores as well as in energy and several macronutrient and micronutrient intake as summarized in Supplementary Tables 4, 5. Egg allergy was not associated with either the DQI-I or nutrient intake (data not shown).

### Associations of DQI-I With Micronutrient Intake

**Figure 5:**
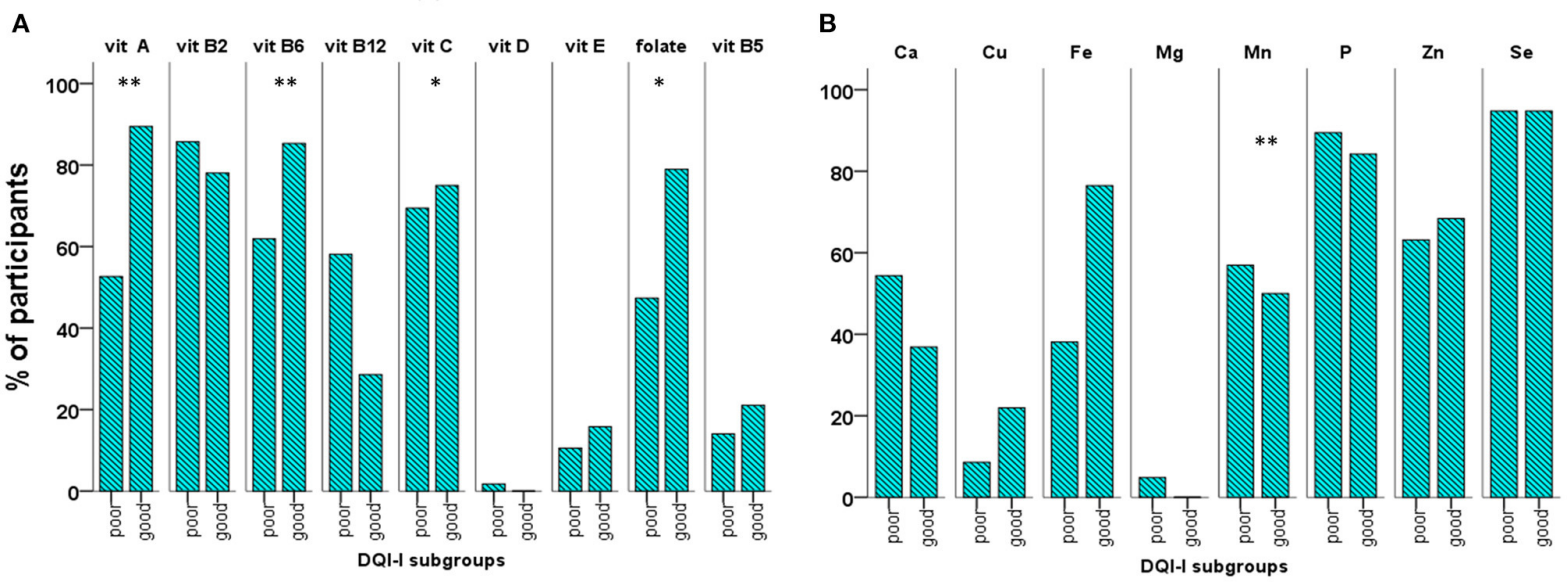
Percentage of participants with vitamin **(A)** and mineral **(B)** intake higher than the reference intake (RI) in the subgroups with poor/good overall quality of diet (good = overall DQI-I =/> 60 points) (*p < 0.05; **p < 0.01).

The overall DQI-I score was significantly, positively correlated with intake of the vitamins A (*p* < 0.001; r 0.479), B3 (*p* = 0.015; r 0.278), B6 (*p* = 0.006; r 0.313), C (*p* < 0.001; r 0.402), E (*p* = 0.001; r 0.389) and folate (*p* < 0.001; r 0.410) as well as with the minerals Cu (*p* < 0.001; r 0.573), Fe (*p* < 0.001; r 0.404), Mg (*p* = 0.002; r 0.351), Mn (*p* < 0.001; r 0.588), and K (*p* = 0.023; r 0.260). Further analysis according the DQI-I subgroups showed that participants with good DQI-I received significantly higher amounts of vitamin A, Cu, and Mn (Table 3), while significantly higher proportions of them received the recommended amounts of vitamins A (*p* = 0.001, B6 (*p* = 0.008), C (*p* = 0.024), folate (*p* = 0.038), and Mn (*p* = 0.010) compared to those with poor DQI-I (Figures 5A,B). No other significant difference between the good and poor DQI-I subgroups regarding vitamin and mineral intake was found.

### Multiple Regression Analysis

#### Factors Independently Associated With the DQI-I and DQI-I Domains

The regression model with independent variables the number of allergies, the age, and sex revealed the number of allergies as a significant independent factor associated with the DQI-I moderation and balance while the age was associated with moderation (Supplementary Table 6). Inclusion of milk avoidance in the model showed that milk avoidance was a significant predictor of the adjusted DQI-I moderation and balance while eliminating the significant association of both the number of allergies and age on DQI-I moderation (Table 4, and Supplementary Table 7).

**Table 4:** Multiple regression analysis: factors independently, significantly associated with the overall DQI-I and DQI-I domains, energy and nutrient intake^*^.

**Dependent variables**	**Independent variables**			
	**Number of allergies**	**Age**	**Sex**	**Milk avoidance**
	**B (*p*)**	**B (*p*)**	**B (*p*)**	**B (*p*)**
DQI-I moderation				−0.201 (**0.012**)
DQI-I balance	−0.186 (**0.005**)			0.469 (**0.043**)
Energy and macronutrient intake				
Energy IR%		−4.614 (**<0.001**)		
Carbohydrate E%		1.699 (**<0.001**)		
Protein E%		0.687 (**0.008**)		
Fat E%		1.013 (**0.014**)		
SFA E%				0.495 (**<0.001**)
Carbohydrates (g/kg/d)		−0.141 (<**0.001**)		
Protein (g/kg/d)		−0.087 (**<0.001**)		
Fat (g/kg/d)		−0.054 (**0.017**)		
Cholesterol (mg/d)		0.071 (**0.050**)		
SFA (g/d)		0.054 (**0.034**)		0.520 (**<0.001**)
Trans (g/d)			0.525 (**0.043**)	
MUFA (g/d)		0.069 (**0.040**)		
PUFA (g/d)		0.068 (**0.027**)		
MUFA:PUFA:SFA		−0.021 (**0.429**)		−0.751 (<**0.001**)
Vitamin intake				
B2	−0.130 (**0.010**)	0.047 (**0.020**)		0.397 (**0.001**)
B3		0.0730 (**0.006**)		
B6	−0.198 (**0.010**)	0.075 (**0.026)**	−0.410 (**0.017**)	−0.553 (**0.004**)
B12		0.045 (**0.193**)		0.372 (**0.045**)
D	−0.384 (**0.002**)			
E	−0.257 (**0.004**)	0.092 (**0.034**)		
Folate			0.375 (**0.022**)	
Mineral intake				
Ca	−0.210 (**0.001**)			0.596 (**<0.001**)
P	−0.093 (**0.049**)			0.288 (**0.010**)
Cu			0.012 (**0.010**)	
K				
Zn				0.290 (**0.011**)
Na	−0.121 (**0.040**)	0.067 (**0.013**)		

* *Only significant associations are shown (all associations are shown in the Supplementary Table 6)*.

*E%, energy as percentage of total energy intake; IR%, intake as percentage of requirements; MUFA, Mono-Unsaturated Fatty Acids; PUFA, Poly-Unsaturated Fatty Acids; SFA, Saturated Fatty Acids. The p-values are shown in bold letters*.

#### Factors Independently Associated With Energy and Macronutrient Intake

The number of allergies showed a significant, negative association with protein E% and cholesterol intake, after controlling for age and sex, which was eliminated following the addition of milk avoidance was added as a factor to the regression model (Table 4 and Supplementary Tables 6, 7). Age was a significant predictor of almost all the assessed macronutrients and energy intake both before and after adjustment for milk avoidance. Milk restriction was significantly associated with saturated fat intake, which was lower in those avoiding milk, and the MUFA:PUFA:SFA balance, that was higher in participants on milk restriction compared to the rest of the study population (Table 4 and Supplementary Table 7).

#### Factors Independently Associated With Micronutrient Intake

The number of allergies had a significant, negative association with vitamins B2, B6, B12, D, and E as well as with Ca, P, and Na intake, after adjustment for age and sex. The age was associated with the adjusted vitamin B1, B2, B3, B12, and B5 (pantothenic acid) intake as well as with Ca, P, K, Na, and Zn intake, while sex was associated only with folate intake (Supplementary Table 5). The addition of milk avoidance in the models changed certain independent associations; the number of allergies was no longer associated with the vitamin B12; age was no longer associated with vitamins B1 and B12 and minerals Ca, P, K, and Zn, but new associations with vitamins B6 and E emerged; milk avoidance was associated with vitamins B2, B6, and B12 as well as with Ca, P, and Zn. Sex was associated with B6, folate, and Cu (Supplementary Tables 4, 6, 7).

## Discussion

In this prospective, observational study of dietary intake in a pediatric population with FA on elimination diet, assessment of diet quality and nutrient intake showed that only a low percentage of children with FA had a good diet quality, as reflected in the DQI-I score, while an even lower proportion enjoyed a balanced diet. Moreover, low proportions of participants had a daily intake of specific micronutrients in accordance with the recommended AI/AR by the EFSA-NDA panel. The number of allergies, age, and avoidance of milk significantly affected the DQI-I moderation and balance, as well as certain macronutrient and micronutrient intake. The DQI-I was significantly correlated with the intake of 11 out of 20 micronutrients assessed. However, analysis based on the DQI-I classification into good and poor DQI-I subgroups showed that only the vitamin A, Cu, and Mn intake was significantly higher in the good DQI-I subgroup. Moreover, the percentage of participants receiving the recommended amounts of micronutrients was higher in the good-DQI-I subgroup than in the poor DQI-I one only regarding the intake of five out of the 20 micronutrients assessed, specifically Mn and vitamins A, B6, C, and folate.

### Overall DQI-I and DQI-I Domains

Previous authors suggested that the DQI-I provides an effective tool for evaluation and cross-national comparison of diet quality in healthy children and adolescents (18, 20). Mean overall DQI-I score in our study cohort (52 points) was marginally lower than the cutoff point of good diet quality indicating an overall moderate diet quality while the proportion of participants with good DQI-I was also low (28%). In agreement with our results, previous studies in healthy children showed a mean score of overall DQI-I ranging between 50 and 57 points (19, 20, 22, 29, 30), and a proportion with good overall DQI-I ranging from 18 to 42% (20, 22, 29, 30). Our findings combined with the reported in healthy children indicate that the overall diet quality in children is low, regardless of the restrictive diets.

Among the DQI-I domains, variety was the most preserved one while moderation and especially balance were the most affected domains. The DQI-I moderation score depends on the intake of total fat, saturated fat, cholesterol, Na, and empty foods, which are related to the development of non-communicable diseases thereby being a measure of the diet quality (29, 30). In contrast to variety and adequacy, high intakes of the nutrients comprising the moderation domain are scored low, as they are associated with increased risk of chronic metabolic and cardiovascular diseases (4). In our cohort, the high mean intake of fat-derived energy (32.7%) exceeded the respective item's cutoff value for perfect score (<20%), contributing to the low moderation score. The low balance score could be attributed to difficulties in attaining a balance among carbohydrates, proteins, and fat as well as between unsaturated and saturated fatty acids. The low mean MUFA:PUFA:SFA ratio (1.2 points) and the low (<30%) proportion of participants meeting the target balance of fatty acids (threshold 1.77 points) have contributed to the low overall diet balance. A similar ratio of MUFA:PUFA:SAT has been found in adults, while a lower ratio was reported in healthy children (1.1 points and 0.2 points, respectively) (29–31). In addition, previous studies in healthy children/adolescents demonstrated low frequency (10–42%) of high PUFA:MUFA:SFA ratio (22, 29, 30).

The agreement of the diet quality found in our study population with the reported in healthy children/adolescents could be attributed to the dietary advice provided by the allergist/gastroenterologist that was efficient in achieving both growth and diet quality comparable to that of healthy children (just 5.3% of the study population were underweight). However, the low overall DQI-I and DQI- moderation and balance scores found in our study and reported in healthy children should be given further consideration. It is important to clarify whether diet quality in children is generally low, regardless of the implementation of restrictive diets, thereby consisting a public health issue, or the DQI-I needs reevaluation as for its application in children and adolescents. Elucidation of these issues could contribute to the implementation of a healthy diet early in childhood and a more precise evaluation of the effect of restrictive diets on diet quality of children with chronic diseases.

### DQI-I Relation With Micronutrient Intake

The DQI-I index calculation does not take into account micronutrient intake, except from Ca, Fe, and vitamin C. Therefore, the association of the DQI-I score and the DQI-I-related subgroups with micronutrient intake was one of the main aims of the current study. Bivariate correlations showed that the DQI-I overall score was significantly, positively correlated with several micronutrient intakes. In line with our results, Williams et al. (19), in a large cohort of pre-adolescents and adolescents (age 10–18 years), found a significant positive association between DQI-I score and nutrient intake. However, further analysis of micronutrient intake by participants with good or poor quality of diet revealed that only the intake of vitamin A, Cu, and Mn was significantly higher in the good-DQI-I subgroup compared to the subgroup with poor DQI-I. Moreover, a considerable percentage of participants with good DQI-I did not receive the recommended amounts of selective minerals and vitamins. These findings suggest that children with FA classified as having a good diet quality based on the DQI-I may still receive insufficient amounts of several micronutrients. Therefore, although the DQI-I is a useful tool for assessing the overall diet quality and detect the domains needing increased awareness, it cannot accurately assess the potentially deficient intake of certain, important micronutrients. In this context, a detailed nutritional analysis of dietary intake using a food dietary records, is an important complementary tool that would help professionals to provide individualized nutritional advice to children.

### Energy and Macronutrient Intake by Children With Food Allergy

Restriction diets, especially diets including foods that are present in many processed foods thereby providing a major bulk of daily nutrients, may severely affect diet variety and adequacy of energy and nutrient intake. Studies by Kim et al. demonstrated that subjects with FA had lower intake of energy, fat, and proteins compared to non-allergic controls (5, 12). In contrast, Flammarion et al. did not find any significant difference in energy and macronutrient intake between allergic children and non-allergic controls (3). This finding was attributed to the fact that 88% of their study population received nutritional counseling by a trained nutritionist. In the lack of control group in our study, we evaluated the adequacy of energy and nutrient intakes in comparison with reference values suggested by EFSA-NDA panel (28). In agreement with findings by Flammarion et al. 99% of our study population received the recommended amount of protein, while <50% received carbohydrate- and fat- derived energy within the reported reference range (3). It is important that more than 1/3 of the study population received high fat E%, indicating a rather unhealthy diet pattern (3). However, the quantitative and qualitative deviation of fat intake from the reference ranges cannot be attributed solely to the FA as similar findings have been reported in heathy children and adolescents, with up to 85% of them receiving high fat-derived E% and 58% having a low unsaturated to saturated fatty acid balance (29).

### Micronutrient Intake by Children With Food Allergy

The intake of vitamins, minerals, and trace elements was lower than the respective reference values in a considerable proportion of our cohort. Our findings are consistent with previous studies showing that children and adolescents with FA on elimination diets may receive inadequate amounts of specific vitamins and minerals depending on the allergens that should be avoided and the supplements added (3, 5, 12, 32). Studies by Kim et al. demonstrated that subjects with FA had lower intake of Ca and vitamin B2 than non-allergic controls (12). In contrast, Flammarion et al. found that children with allergy had higher intake of vitamin D and E, which was attributed to the supplemental oil recommended by the nutritionist (3). Low intake of vitamin D was a common finding of previous studies in allergic children (5, 32, 33). This is not surprising as studies in a general European population demonstrated that vitamin D intake was lower than the recommended in most children and adults (32, 34). These reports align with the very low proportions of our study population meeting the daily vitamin D requirements, while only half of them met the recommended Fe and Ca intake. However, in contrast to previous studies, a considerable proportion of our study population received adequate amounts of vitamin B2 (82%) and C (70%).

The use of different reference values may have contributed to the differences among studies. In the current study, the AI and AR recommended by the EFSA-NDA panel were used for the evaluation of adequate intake. The EFSA-NDA panel stated that “the Average Requirement can be used to estimate the prevalence of inadequate intakes of micronutrients (the Average Requirement cut-point method), if the distribution of nutrient intakes is normal, and intakes are independent from requirements” (28). In the context of the non-Gaussian distribution of values found in our study, we focused on the proportions of participants with intake within the reference range or above the AI / AR rather than those with low intakes. Moreover, values lower than the reference intake do not necessarily indicate micronutrient insufficiency (35, 36). Assessment of serum levels of micronutrients and nutrition biomarkers, could aid to a more precise evaluation regarding the sufficiency of micronutrient intake in children with FA (37, 38).

### Effect of Multiple Allergies

The reported prevalence of multiple food allergies ranged from 19 to 96% (5, 35, 39–42). In the current study, about half of the participants had multiple food allergies, with 28% being sensitive to two foods, in line with the reported frequency of two-food allergies (around 30% of total cases) (35, 39, 41, 42). Multiple allergies may further compromise diet quality and certain micronutrient intake (43). In our study, the number of FA was positively associated with the DQI-I moderation and balance, which, surprisingly, were higher in patients with multiple allergies. These associations could be attributed to a higher compliance of children allergic to multiple foods with dietary instructions due to increased awareness of allergic symptoms. The significant association of DQI-I moderation with the number of allergies disappeared after adjustment for milk avoidance indicating that milk has a stronger association with diet moderation than the number of allergies.

Reported data regarding the effect of the number of FA on nutrient intake is controversial. A systematic review of studies in children with IgE-mediated multiple FA concluded that multiple allergies may be associated with increased risk of inadequate nutrient intake compared to children without FA (10). Similarly, Christie et al. reported that significantly higher proportions of children with multiple allergies received Ca lower than the recommended intake (5). In contrast, Flammarion et al. did not find any difference in macronutrient intake between children with multiple and single allergies (3). In our study, the total energy and the specific macronutrient – derived energy were not affected by the number of allergies. Regarding micronutrient intake, only Ca and vitamin D intake were significantly lower in participants with multiple allergies on bivariate analysis. However, adjustment for age, sex, and milk avoidance revealed that the number of FA was negatively associated with the intake of vitamins B2, B6, D, and E, Ca, and P. Collectively, these findings suggest that children with multiple FA are at increased risk for micronutrient insufficiency.

### Effect of Age

The age did not have any significant impact on the unadjusted and adjusted overall DQI-I. In contrast, Mariscal et al. reported a significant effect of age on overall DQI-I score, with younger children having higher DQI-I scores compared to adolescents (29). The different results may be due to the higher age of the population (6–18 years) included in the previous study compared to the current study. Further analysis in relation to DQI-I domains, showed that schoolchildren had higher variety, as expected, but lower moderation compared to toddlers. These age-related differences could be attributed to a stricter parental control on toddlers' diet consuming almost exclusively homemade foods.

Adequacy of nutrient intake may vary with age due to changes in nutrient requirements with advancing age (38, 44). In our study, age was a significant factor independently associated with most macronutrient and micronutrient intake before and after adjustment for cofactors. The positive association of age with the adjusted fat- and SFA- derived energy as well as the trans fatty acid intake are indicative of a deterioration of diet quality with advancing age of children with FA. Adjustment for either milk or egg avoidance did not change significantly the above associations indicating that the effect of age is stronger than the effect of the kind of food avoided. These changes further support the view that schoolchildren are more often exposed to junk foods rich in total fat, saturated fat, and trans fatty acids, and potentially allergenic ingredients.

Concerning the effect of age on micronutrient intake, increased age was associated with increased daily intake of vitamin B2, vitamin B12, Ca, P, Zn, and Na by our study population. However, comparison with the reference AI/AR revealed that a lower proportion of schoolchildren attained the reference intake of Fe, Mn, K, and folate compared to toddlers. The significant independent association of age with micronutrient intake remained after adjustment for the number of allergies and sex. These findings emphasize the importance of periodic assessment of diet quality and nutrient intake, as recommended by the joint Task Force of the Italian Society of Pediatric Nutrition (SINUPE) and the Italian Society of Pediatric Allergy and Immunology (SIAIP) (38).

### Effect of the Foods Avoided

The effect of FA on nutritional intake depends on the specific food avoidance. In line with previous studies, cow's milk and egg, which are used as ingredients in many processed foods, were the most common food allergens (41, 45). Therefore, we further analyzed our results in association with milk or egg avoidance. Milk provides energy and protein, Ca, P, Mg, Zn, and vitamins B2 and B12, while it is often fortified with vitamins A and D (46). Milk-free diet has been associated with low Fe, Ca, Zn, and vitamin B2, C, D, and E intake (4, 12, 47–51). We found that participants avoiding milk were younger and received significantly lower daily amounts of specific micronutrients, including Ca and vitamin D, compared to the rest of the study population. In the context of the significantly lower age of the participants that avoided milk and the associations of age with the DQI-I and nutrient intake, we added milk avoidance in the regression models as an independent factor. This analysis revealed milk avoidance as a significant factor associated with diet moderation (inversely) and balance (positively), indicating that participants with milk allergy had higher diet moderation but lower balance. In addition, milk avoidance was an independent predictor of Ca, P, Zn, and vitamins B2, B6, and B12 intake. In fact, inclusion of milk avoidance in the regression models eliminated the association of age with the DQI moderation and changed the associations with several nutrients, indicating a strong effect of milk avoidance on DQI-I moderation and nutrient intake. The strong impact of milk allergy on micronutrient intake should be taken into consideration when recommending nutritional supplements to allergic children.

Egg is a source of vitamins B2, B5 (pantothenic acid), B7 (biotin), and B12, and selenium (38). Egg elimination diets have been associated with low intake of vitamins A, B1, B2, and B3 (niacin) (12). Unlike reported data, our results did not show any association of egg avoidance with the unadjusted and adjusted values of diet quality and micronutrient intake. The difference from the previous study may be attributed to improved dietary management of FA over time.

### Limitations and Strengths

The main limitation of the study is the lack of control group that would allow comparison of our findings in children with FA with those in their healthy peers. Instead, we evaluated the quality of diet and adequacy of nutrient intake according to the published reference values/range for good quality of diet and the age- and sex- specific reference intakes (EFSA) (28). In the context of the different reference values used in relevant studies, comparison between studies regarding the adequacy of nutrient intake by children with FA should be interpreted with caution. Nevertheless, despite any differences in the reference values utilized, most studies, including the current one, indicate that FA, especially the multiple allergies, may compromise the moderation and balance of diet and certain nutrient intake, depending on the kind of food avoided and the age. Therefore, it is highly recommended that nutritional counseling by a trained nutritionist tailored for the individual nutritional requirements should be offered for every child with FA.

Another limitation is the fact that the DQI-I tool has been developed for healthy children. Although the tool has been already used for other pathologic pediatric conditions (23, 24), our results would have been more representative if a diet quality tool specifically designed for children with FA was used. To our knowledge no such tool has been developed as yet. Moreover, the DQI-I has not been validated for micronutrients (17, 29). To overcome this limitation, we analyzed the data as for individual nutrient intake, in addition to DQI-I. Finally, the sample size was not large enough to enable analysis of DQI-I and nutrient intake in relation to more food allergens.

The current study is the first to report the effect of elimination diets on the overall diet quality of children with FA using the DQI-I tool and evaluate the capability of DQI-I to assess the adequacy of individual nutrient intake in this vulnerable population. Another strength of our study is the nutrition assessment both with a 24-h dietary recall tool and a 7-day FFQ, accurately depicting food intake. In addition, the dietary assessment was performed by a trained nutritionist through interview of either parents/caregivers or the children themselves during their visit to the outpatient clinic in order to eliminate response bias.

## Conclusions

Children with FA undergoing elimination diets have low diet quality characterized by low moderation and balance. However, our findings combined with previous reports in healthy children, showing a similarly low diet quality assessed by the DQI-I, indicate that even though children with FA may be on increased risk of low diet quality due to avoidance of specific food products or groups, low diet quality seems to be a concern and a public health issue also for the general pediatric population. In the context of the increased vigilance shown by the parents of children with FA as for their children's dietary habits and nutrition, the dietary patterns of the general pediatric population rather than the elimination diets may be the main reason for low diet quality. DQI-I was found to be accurate in capturing the deflection of diet quality related to the development of chronic, non-communicable diseases through its moderation and balance components. This is the DQI-I's main purpose as a healthy diet indicator and it would be a useful tool responding to the needs of the contemporary shifting of priorities in FA-children's diet from quantity to quality. Nevertheless, it does not accurately reflect the intake of certain micronutrients that can be compromised by elimination diets. Therefore, regular nutritional assessment utilizing both the DQI-I and tools assessing individual nutrient intakes as well as counseling by a trained professional should be integral parts of the individualized management of children with FA. Proper elimination diets should be carefully implemented, taking into account the number of allergies, age, and food avoidance, in order to ensure not only an adequate nutrient intake but also a high quality and healthy eating patterns. Development of a tool specifically adapted to accurately assess the diet quality of children with FA would facilitate the design of strategies and research aiming at improving diet quality in this vulnerable pediatric population.

## Data Availability Statement

The raw data supporting the conclusions of this article will be made available by the authors, without undue reservation.

## Ethics Statement

The studies involving human participants were reviewed and approved by Thomai Karagiozoglou-Lampoudi, Menelaos Zafrakas, and Georgios Bambidis, Bioethics committee Alexandrion Technological Education Institute (currently: International Hellenic University), Thessaloniki, Greece. Written informed consent to participate in this study was provided by the participants' legal guardian/next of kin.

## Author Contributions

TK-L, IX, CA, and AM conceptualized the study. AK was responsible for nutrition assessment, dietary intake analysis, and DQI-I calculation. IX was responsible for the pediatric gastroenterology outpatient clinic, evaluated, treated, and followed up the patients and supervised data collection. CA performed data analysis, contributed to the interpretation of the results, and drafted the manuscript. AM was responsible for the pediatric allergology outpatient clinic, evaluated, treated, and followed up the patients. DM, AT, and EA assisted patient evaluation and contributed to data collection and manuscript preparation. TK-L was responsible for the study design, data collection planning, and the interpretation of the results. All authors critically reviewed the manuscript and approved the final version of the manuscript for submission.

## Conflict of Interest

The authors declare that the research was conducted in the absence of any commercial or financial relationships that could be construed as a potential conflict of interest.

## Supplementary Material

Supplementary Figure 1 Box plots of the overall DQI-I and DQI-I domain points in participants with poor (<60% of perfect score) and good diet quality. ^**^*p* < 0.01; ^***^*p* < 0.001.

Supplementary Figure 2 Percentage of participants with overall DQI-I and DQI-I domain score higher than or equal to 60% of the perfect score in age–related subgroups. There was no significant difference.

Supplementary Figure 3 Percentage of participants receiving adequate, excess, or low total energy (IR%, intake as percentage of requirements), carbohydrate- and fat-derived E% (energy as percentage of total daily energy), and protein (g/kg/d) in the age–related subgroups. ^**^*p* < 0.01.

Supplementary Figure 4 Percentage of participants with vitamin intake higher than the reference intake (RI) in age–related subgroups. ^**^*p* < 0.01.

Supplementary Figure 5 Percentage of participants with mineral intake higher than the reference intake (RI) in age–related subgroups. ^*^*p* < 0.05; ^**^*p* < 0.01.

Supplementary Table 1 *post-hoc* analysis of the power for detecting significant differences of the DQI-I and DQI-I domain scores between the subgroups related to DQI-I categories, number of allergies, age, and milk avoidance.

Supplementary Table 2 Overall DQI-I points, DQI-I domain points, energy intake, and macronutrient intake in the sex – related subgroups.

Supplementary Table 3 Micronutrient intake (median, interquartile range) in the sex – related subgroups.

Supplementary Table 4 Overall DQI-I and domain points, energy and macronutrient intake in relation to the avoided foods.

Supplementary Table 5 Micronutrient intake (median, interquartile values) in relation to the foods that were avoided.

Supplementary Table 6 Multiple regression analysis Model 1: Factors independently associated with the overall DQI-I, DQI-I domains, and nutrient intake.

Supplementary Table 7 Multiple regression analysis Model 2: Factors independently associated with the overall DQI-I and DQI-I domains, energy and nutrient intake.

## Abbreviations

AI: adequate intake
AR: average requirements
DQI-I: Diet Quality Index – International
E%: energy as percentage of energy intake
EFSA: European Food Safety Authority
FA: Food Allergy
FFQ: Food Frequency Questionnaire
IR%: Intake as percentage of Requirements
MUFA: Mono-Unsaturated Fatty Acids
NDA, Dietetic Products: Nutrition and Allergies
PUFA: Poly-Unsaturated Fatty Acids
SFA: Saturated Fatty Acids.
