# Supplementary figures and images for: Diet Quality: A Neglected Parameter in Children With Food Allergies. A Cross–Sectional Study
Source: Front Pediatr. 2021 Apr 23;9:658778. doi: 10.3389/fped.2021.658778 (PMC8102985; doi:10.3389/fped.2021.658778)

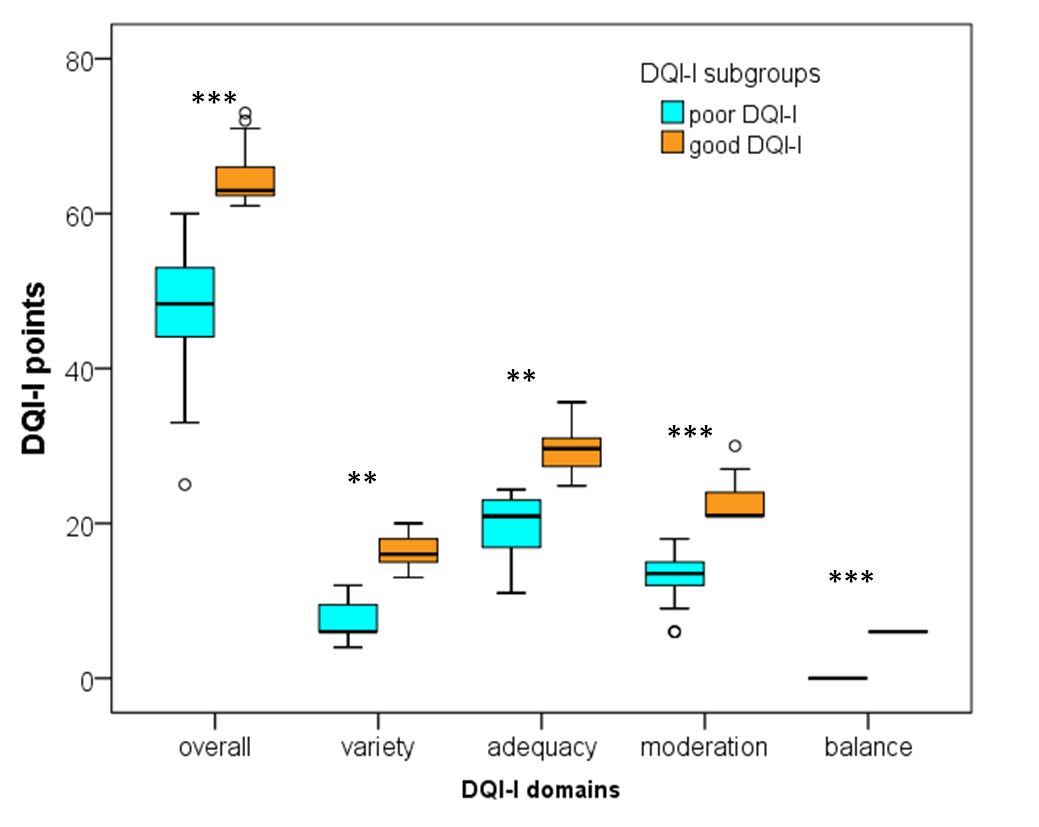

Supplement: Supplementary Figure 1 — Box plots of the overall DQI-I and DQI-I domain points in participants with poor (<60% of perfect score) and good diet quality. **p < 0.01; ***p < 0.001. [file Image_1.tif]

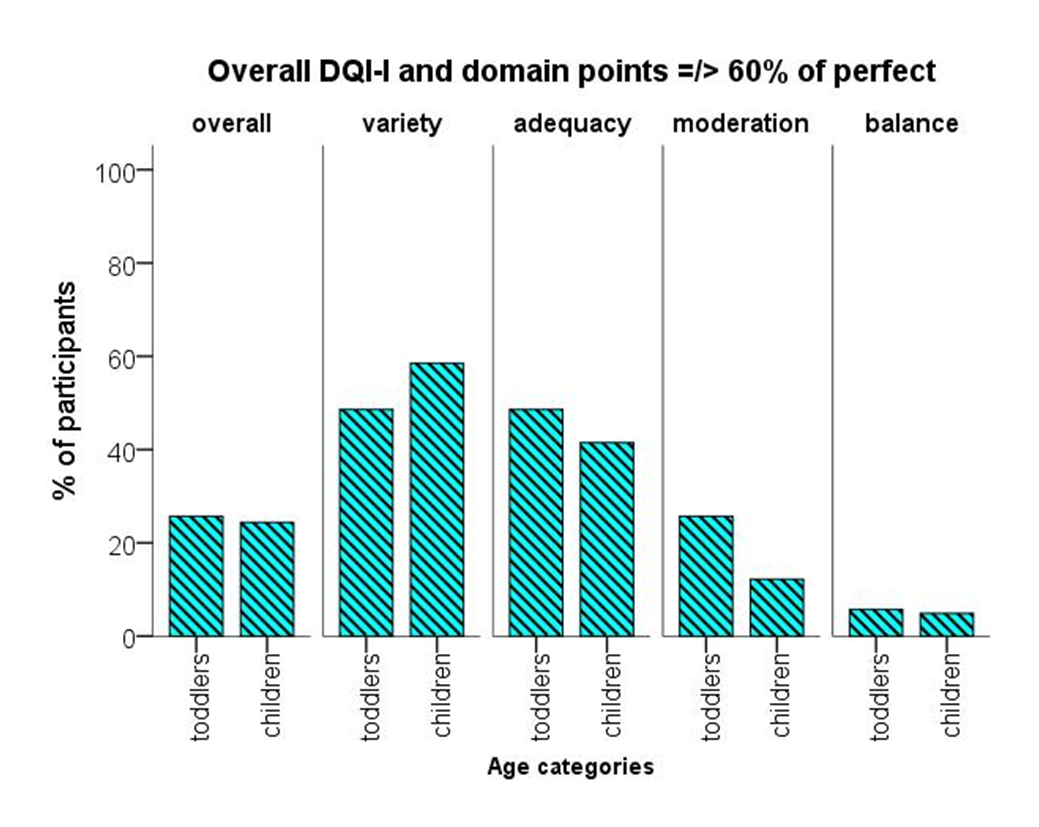

Supplement: Supplementary Figure 2 — Percentage of participants with overall DQI-I and DQI-I domain score higher than or equal to 60% of the perfect score in age–related subgroups. There was no significant difference. [file Image_2.tif]

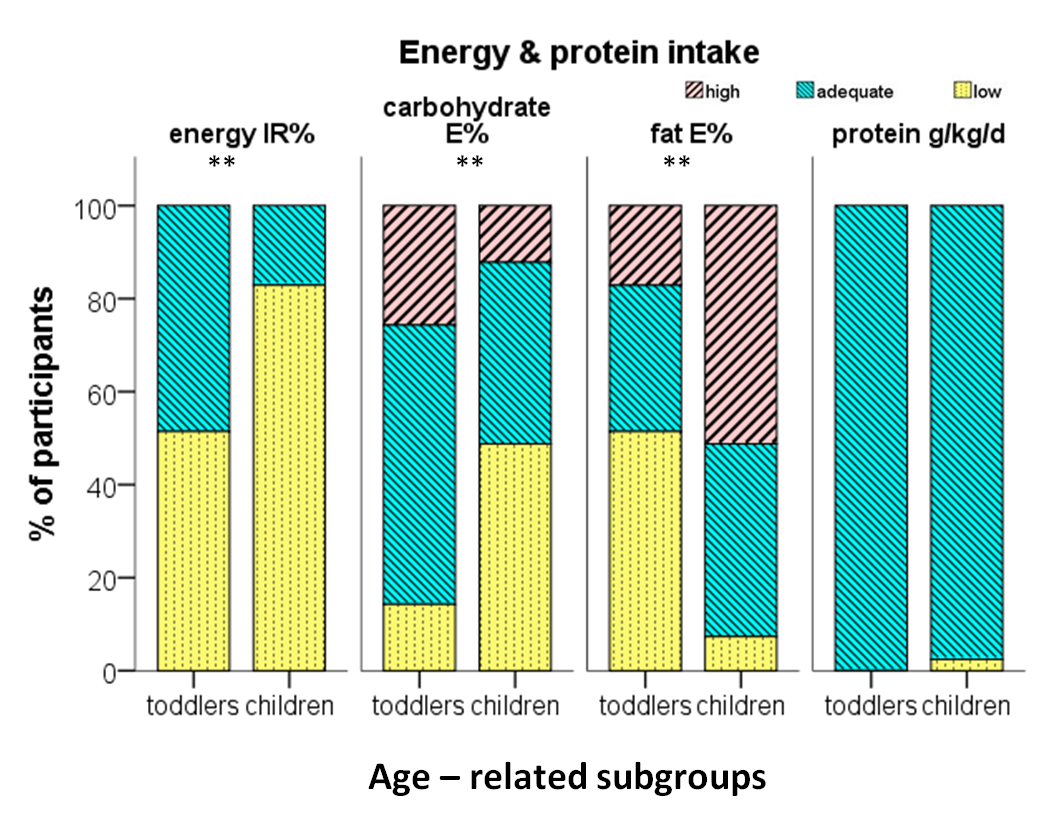

Supplement: Supplementary Figure 3 — Percentage of participants receiving adequate, excess, or low total energy (IR%, intake as percentage of requirements), carbohydrate- and fat-derived E% (energy as percentage of total daily energy), and protein (g/kg/d) in the age–related subgroups. **p < 0.01. [file Image_3.tif]

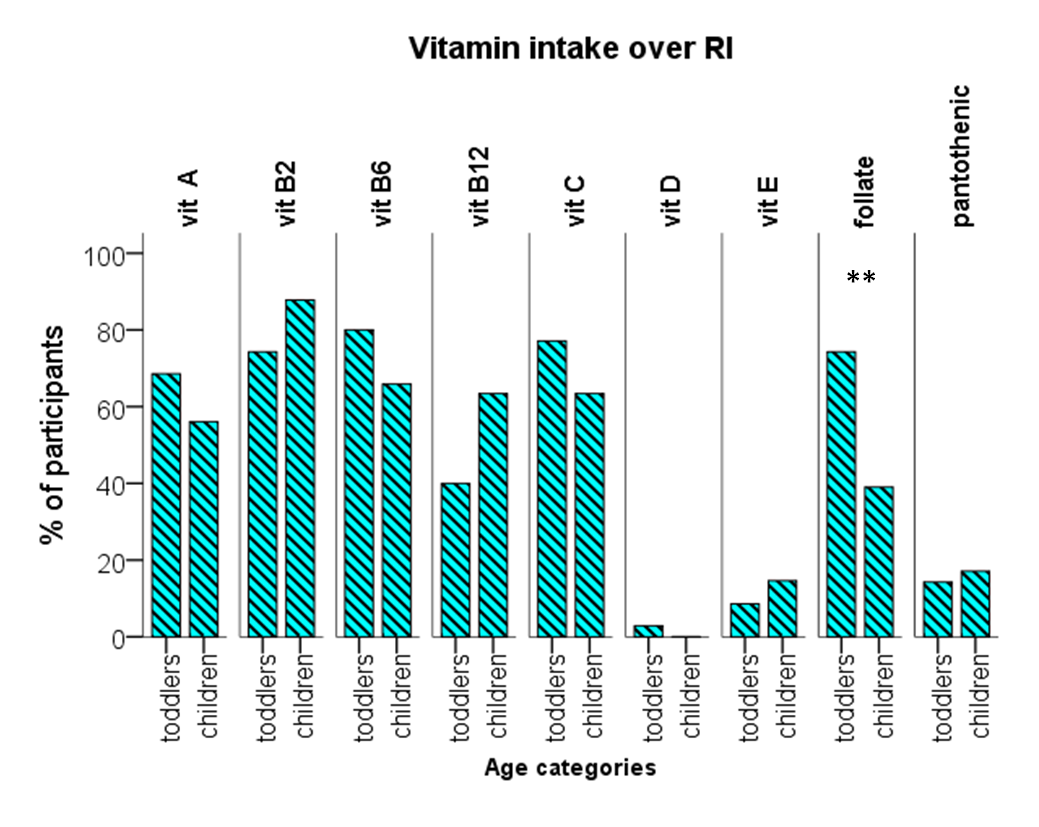

Supplement: Supplementary Figure 4 — Percentage of participants with vitamin intake higher than the reference intake (RI) in age–related subgroups. **p < 0.01. [file Image_4.tif]

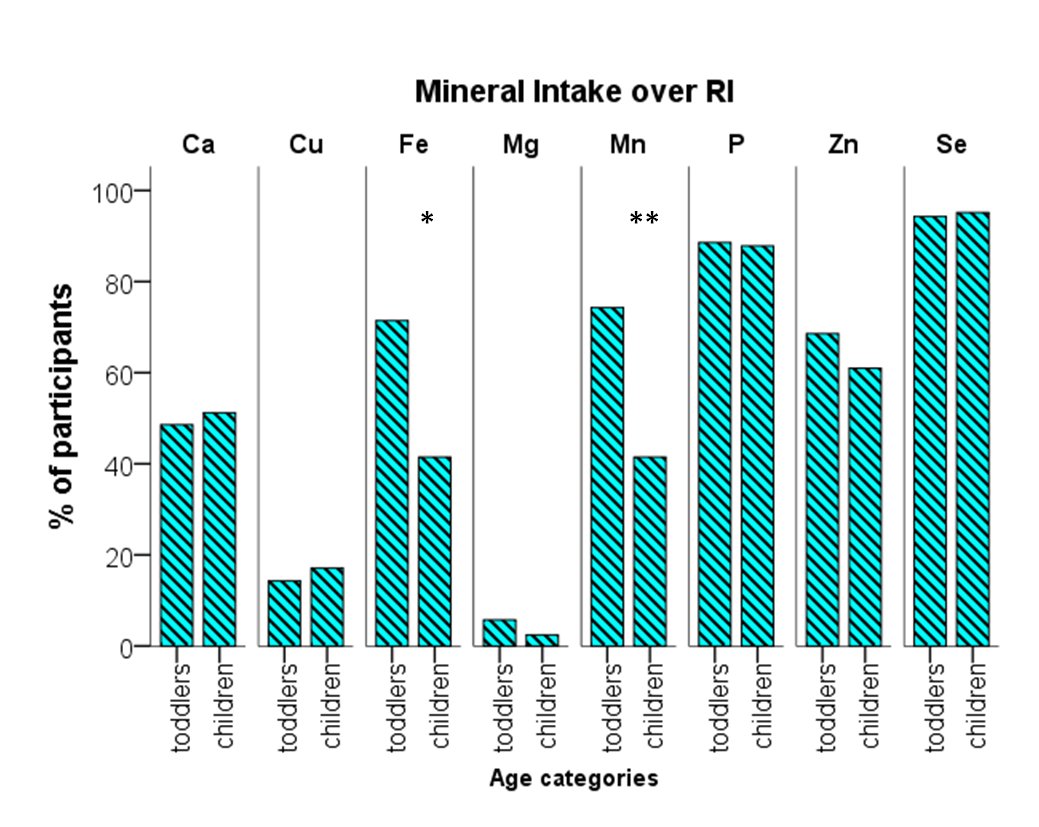

Supplement: Supplementary Figure 5 — Percentage of participants with mineral intake higher than the reference intake (RI) in age–related subgroups. *p < 0.05; **p < 0.01. [file Image_5.tif]
