# Supplementary material for: Diet Quality: A Neglected Parameter in Children With Food Allergies. A Cross–Sectional Study
Source: Front Pediatr. 2021 Apr 23;9:658778. doi: 10.3389/fped.2021.658778 (PMC8102985; doi:10.3389/fped.2021.658778)
Supplement: Supplementary Table 1 — post-hoc analysis of the power for detecting significant differences of the DQI-I and DQI-I domain scores between the subgroups related to DQI-I categories, number of allergies, age, and milk avoidance. [file Table_1.docx]

SUPPLEMENTARY FILE

Supplementary Table 1. Post-hoc analysis of the power for detecting significant differences of the DQI-I and DQI-I domain scores between the subgroups related to DQI-I categories, number of allergies, age, and milk avoidance

|  | Poor vs. good DQI-I | Single vs. multiple allergies | Toddlers vs. children | Milk avoidance Yes vs. No |
| --- | --- | --- | --- | --- |
| N | % | % | % | % |
| Overall DQI-I | 100 | 18.4 | 6.35 | 47.6 |
| DQI-I variety | 89.6 | N.A.* | 46.6 | 19.7 |
| DQI-I adequacy | 47 | 18.8 | 27 | 8.2 |
| DQI-I moderation | 100 | 67.9 | 54.3 | 98.9 |
| DQI-I balance | 76.5 | 16.8 | 9.8 | 29.7 |

N.A. not applicable due to identical mean values

Supplementary Table 2. Overall DQI-I points, DQI-I domain points, energy intake, and macronutrient intake in the sex – related subgroups

|  | Male | Female | p |
| --- | --- | --- | --- |
| N | 28 | 48 |  |
| Number of allergies (mean ± SD) | 1.79 ± 1.3 | 1.73 ± 1.0 | 0.743 |
| Age (years) | 5.0 (5.0) | 4.2 (3.4) | 0.408 |
| Overall DQI-I (mean ± SD) | 52.4 ± 10.7 | 51.9 ± 9.6 | 0.959 |
| DQI-I variety (mean ± SD) | 10.9 ± 5.0 | 13.1 ± 4.5 | 0.070 |
| DQI-I adequacy (mean ± SD) | 24.7 ± 6.1 | 23.6 ± 6.1 | 0.314 |
| DQI-I moderation (mean ± SD) | 15.2 ± 4.7 | 14.6 ± 5.1 | 0.557 |
| DQI-I balance (mean ± SD) | 0.79 ± 1.6 | 0.92 ± 1.8 | 0.910 |
| Carbohydrate (g/kg/d; median [IQR]) | 8.1 (6.9) | 8.3 (7.2) | 0.909 |
| Protein (g/kg/d; median [IQR]) | 2.4 (1.7) | 2.4 (1.2) | 0.832 |
| Fat (g/kg/d; median [IQR]) | 1.9 (1.6) | 2.3 (1.0) | 0.164 |
| Energy IR % (mean ± SD) | 85.3 ± 29.7 | 87.8 ± 30.8 | 0.851 |
| Carbohydrate E% (mean ± SD) | 52.4 ± 12.8 | 49.2 ± 13.5 | 0.433 |
| Protein E% (mean ± SD) | 17.0 ± 5.5 | 17.1 ± 6.4 | 0.994 |
| Fat E% (mean ± SD) | 30.6 ± 9.7 | 33.8 ± 9.9 | 0.282 |
| SFA E% (mean ± SD) | 11.5 (9.1) | 11.1 (11.6) | 0.936 |
| Trans fatty acids (g/d; median [IQR]) | 0.58 (1.1) | 0.46 (0.52) | 0.726 |
| MUFA (g/d; median [IQR]) | 12.6 (12.6) | 13.4 (11.8) | 0.436 |
| PUFA (g/d; median [IQR]) | 4.6 (4.1) | 4.4 (4.5) | 0.730 |
| MUFA:PUFA:SAT (median [IQR]) | 1.2 (1.0) | 1.2 (1.2) | 0.979 |
| Omega 3 (g/d; median [IQR]) | 0.32 (0.33) | 0.36 (0.24) | 0.714 |
| Omega 6 (g/d; median [IQR])) | 2.7 (2.6) | 2.9 (3.7) | 0.458 |
| Cholesterol (g/d; median [IQR]) | 119 (132) | 120 (150) | 0.357 |

E%, energy as percentage of total energy intake; IQR, interquartile range; IR%, intake as percentage of requirements; MUFA, Mono-Unsaturated Fatty Acids; PUFA, Poly-Unsaturated Fatty Acids; SFA, Saturated Fatty Acids.

Supplementary Table 3. Micronutrient intake (median, interquartile range) in the sex – related subgroups

|  | Male | Female | p |
| --- | --- | --- | --- |
| N | 28 | 48 |  |
| vit A (μg/d) | 478.5 (1020) | 504 (628) | 0.423 |
| Vit B1 (mg/MJ) | 0.91 (0.5) | 0.75 (0.7) | 0.738 |
| Vit B2 (mg/d) | 1.0 (0.6) | 1.2 (1.1) | 0.455 |
| Vit B3 (mg/MJ) | 9.7 (5.8) | 8.7 (7.4) | 0.282 |
| Vit B6 (mg/d) | 0.81 (0.6) | 0.95 (0.9) | 0.540 |
| Vit B12 μg/d) | 1.9 (1.7) | 2.1 (2.4) | 0.561 |
| Vit C (mg/d) | 35.9 (73) | 41.8 (61) | 0.517 |
| Vit D (μg/d) | 2.7 (4) | 3.1 (5) | 0.663 |
| Vit E (mg/d) | 2.3 (2.7) | 2.7 (3.0) | 0.609 |
| Folate (μg/d) | 154 (122) | 122 (72) | 0.049 |
| Pantothenic acid (mg/d) | 2.5 (1.2) | 2.5 (1.4) | 0.714 |
| Ca (mg/d) | 550 (495) | 602 (671) | 0.296 |
| Cu (mg/d) | 0.58 (0.37) | 0.57 (0.48) | 0.617 |
| Fe (mg/d) | 6.8 (3.89) | 6.3 (4.0) | 0.580 |
| Mg (mg/d) | 142 (71) | 140 (77) | 0.634 |
| Mn (mg/d) | 0.9 (1.1) | 1.1 (1.0) | 0.934 |
| P (mg/d) | 701 (423) | 700 (440) | 0.714 |
| K (mg/d) | 1578 (620) | 1519 (669) | 0.970 |
| Se (µg/d) | 59 (59) | 59 (60) | 0.919 |
| Zn (µg/d) | 5.8 (3.5) | 5.4 (5.0) | 0.783 |

Supplementary Table 4. Overall DQI-I and domain points, energy and macronutrient intake in relation to the avoided foods

|  | Foods avoided | | | | | |
| --- | --- | --- | --- | --- | --- | --- |
|  | Milk avoidance | | | Egg avoidance | | |
|  | NO | YES | p | NO | YES | p |
| N | 36 | 40 |  | 40 | 36 |  |
| DQI-I overall points | 49.9 ± 9.1 | 54.1 ± 10.4 | 0.039 | 51.8 ± 9.3 | 52.5 (14.7) | 0.369 |
| DQI-I variety points | 12.9 ± 5.2 | 11.7 ± 4.3 | 0.218 | 12.2 ± 5.1 | 12.3 ± 4.4 | 0.869 |
| DQI-I adequacy points | 23.6 ± 6.5 | 24.4 ± 5.7 | 0.735 | 24.1 ± 6.4 | 21.9 ± 5.8 | 0.879 |
| DQI-I moderation points | 12.6 ± 3.7 | 16.6 ± 5.1 | <0.001 | 14.5 ± 4.6 | 15.2 ± 5.3 | 0.694 |
| DQI-I balance points | 0.56 ± 1.6 | 1.1 ± 1.7 | <0.001 | 0.9 ± 1.9 | 0.8 ± 1.5 | 0.774 |
| Poor DQI-I (n, %) | 29 (52.7) | 26 (65.0) | 0.199 | 31 (77.5) | 24 (66.7) | 0.316 |
| Good DQI-I (n, %) | 7 (33.3) | 14 (35.0) |  | 9 (22.5) | 12 (33.3) |  |
| Energy IR% | 74.1 (36.2) | 94.2 (35.1) | 0.004 | 87.4 (34.1) | 84.0 (42.7) | 0.668 |
| Carbohydrate E% | 43.6 (17.2) | 53.9 (14.6) | 0.005 | 50.1 (24.4) | 51.8 (19.7) | 0.655 |
| Protein E% | 18.3 (6.6) | 13.5 (7.3) | 0.001 | 15.0 (8.8) | 16.3 (8.6) | 0.750 |
| Fat E% | 36.2 (16.0) | 32.2(12.3) | 0.015 | 33.8 (13.4) | 33.6 (12.7) | 0.655 |
| SFA E% | 16.4 (10.6) | 9.0 (7.6) | <0.001 | 11.8 (9.4) | 10.9 (9.9) | 0.616 |
| Carbohydrate (g/kg/d) | 5.4 (4.6) | 10.8 (5.1) | <0.001 | 8.3 (7.2) | 8.1 (6.7) | 0.808 |
| Protein (g/kg/d) | 2.2 (1.5) | 2.6 (1.5) | 0.381 | 1.8 (1.3) | 2.6 (1.7) | 0.227 |
| Fat (g/kg/d) | 2.0 (1.1) | 2.3 (1.5) | 0.095 | 2.1 (1.5) | 2.2 (1.5) | 0.298 |
| SFA (g/d) | 22.1 (16.8) | 11.9 (8.0) | <0.001 | 14.7 (13.3) | 14.5 (15.8) | 0.776 |
| MUFA (g/d) | 15.0 (13.1) | 12.8 (8.9) | 0.719 | 13.2 (12.3) | 12.6 (11.9) | 0.916 |
| PUFA (g/d) | 4.2 (4.4) | 4.5 (4.6) | 0.651 | 4.5 (3.9) | 4.4 (4.5) | 0.647 |
| PUFA:MUFA:SFA balance | 0.85 (0.7) | 1.7 (1.6) | <0.001 | 1.1 (1.1) | 1.4 (1.1) | 0.675 |
| Cholesterol (mg/d) | 148 (133) | 81 (136) | 0.006 | 138 (137) | 108 (162) | 0.268 |
| Omega 3 (g/d) | 0.45 (0.3) | 0.26 (0.2) | <0.001 | 0.34 (0.2) | 0.32 (3) | 0.480 |
| Omega 6 (g/d) | 2.8 (3.3) | 2.7 (3.7) | 0.632 | 2.7 (3.3) | 2.9 (3.4) | 0.814 |

Values are expressed as median (interquartile range), unless otherwise stated; E%, energy as percentage of total energy intake; IR%, intake as percentage of requirements; MUFA, Mono-Unsaturated Fatty Acids; PUFA, Poly-Unsaturated Fatty Acids; poor DQI-I, DQI-I below 60% of perfect; SFA, Saturated Fatty Acids.

Supplementary Table 5. Micronutrient intake (median, interquartile values) in relation to the foods that were avoided

|  | Foods avoided | | | | | |
| --- | --- | --- | --- | --- | --- | --- |
|  | Milk avoidance | | | Egg avoidance | | |
|  | NO | YES | p | NO | YES | p |
| N | 36 | 40 |  | 40 | 36 |  |
| Age (years) | 7.1 (4.0) | 3.4 (2.0) | < 0.001 | 5.5 (5.8) | 4.0 (4.4) | 0.590 |
| Vit A (μg/d) | 281 (656) | 529 (834) | 0.159 | 462 (763) | 495 (661) | 0.696 |
| Vit B1 (mg/MJ) | 0.95 (0.84) | 0.72 (0.38) | 0.122 | 0.79 (0.52) | 0.83 (0.67) | 0.369 |
| Vit B2 mg/d | 1.4 (0.7) | 0.8 (0.7) | <0.001 | 1.2 (0.5) | 0.9 (1.2) | 0.658 |
| Vit B3 (mg/MJ) | 9.6 (7.9) | 8.9 (7.4) | 0.366 | 9.0 (1.2) | 10.2 (5.6) | 0.503 |
| Vit B6 (mg/d) | 0.9 (0.9) | 0.9 (0.7) | 0.655 | 0.8 (0.6) | 1.0 (0.9) | 0.723 |
| Vit B12 (μg/d) | 2.7 (1.5) | 1.2 (1.4) | <0.001 | 2.0 (1.8) | 2.0 (2.6) | 0.948 |
| Vit C (mg/d) | 39 (48) | 40 (76) | 0.359 | 47 (64) | 37 (81) | 0.690 |
| Vit D (μg/d) | 4.0 (8.2) | 0.67 (3.7) | <0.001 | 3.1 (4.4) | 1.5 (4.7) | 0.190 |
| Vit E (mg/d) | 2.0 (3.1) | 3.0 (2.4) | 0.331 | 2.5 (3.0) | 2.5 (2.8) | 0.766 |
| Folate (μg/d) | 122 (125) | 114 (71) | 0.518 | 126 (84) | 113 (109) | 0.776 |
| Pantothenic acid (mg/d) | 9.9 (1.4) | 2.2 (1.4) | 0.004 | 2.6 (1.1) | 2.5 (1.8) | 0.394 |
| Ca (mg/d) | 781 (489) | 328 (439) | < 0.001 | 602 (428) | 552 (733) | 0.518 |
| Cu (mg/d) | 0.56 (0.5) | 0.59 (0.3) | 0.625 | 0.53 (0.4) | 0.66 (0.5) | 0.321 |
| Fe (mg/d) | 7.4 (5.3) | 6.2 (3.0) | 0.180 | 6.0 (4.2) | 7.0 (3.7) | 0.647 |
| Mg (mg/d) | 168 (86) | 137 (76) | 0.063 | 137 (69) | 143 (92) | 0.504 |
| Mn (mg/d) | 1.0 (1.0) | 1.1 (1.0) | 0.981 | 1.1 (1.0) | 1.1 (1.1) | 0.850 |
| P (mg/d) | 832 (380) | 552 (324) | <0.001 | 695 (358) | 710 (601) | 0.903 |
| K (mg/d) | 1581 (990) | 1445 (670) | 0.039 | 1493 (474) | 1529 (872) | 0.698 |
| Se (µg/d) | 50.0 (62.2) | 52.5 (56.3) | 0.808 | 52.9 (50.8) | 51.2 (65.5) | 0.905 |
| Na (mg/d) | 1171 (878) | 710 (572) | 0.001 | 1009 (999) | 772 (750) | 0.235 |
| Zn (µg/d) | 7.2 (5.4) | 4.8 (3.3) | 0.001 | 5.4 (3.4) | 5.9 (5.9) | 0.519 |

Supplementary Table 6. Multiple regression analysis Model 1: Factors independently associated with the overall DQI-I, DQI-I domains, and nutrient intake

| Dependent variables | Independent variables | | |
| --- | --- | --- | --- |
|  | Number of allergies | Age (years) | sex |
|  | B (p) | B (p) | B (p) |
| DQI-I | 0.015 (0.464) | - 0.012 (0.115) | 0.016 (0.734) |
| DQI-I variety | - 0.019 (0.664) | 0.017 (0.333) | - 0.178 (0.067) |
| DQI-I adequacy | - 0.037 (0.188) | - 0.010 (0.326) | 0.058 (0.355) |
| DQI-I moderation | 0.072 (0.031) | - 0.43 (< 0.001) | 0.070 (0.335) |
| DQI-I balance | - 0.239 (< 0.001) | 0.030 (0.458) | - 0.016 (0.931) |
| Energy and macronutrient intake | | | |
| Energy IR% | 0.058 (0.076) | - 0.059 (< 0.001) | - 0.006 (0.935) |
| Carbohydrate E% | 0.023 (0.367) | - 0.039 (< 0.001) | 0.089 (0.117) |
| Protein E% | - 0.069 (0.045) | 0.042 (0.001) | - 0.039 (0.602) |
| Fat E% | 0.004 (0.901) | 0.038 (0.003) | - 0.114 (0.127) |
| SFA E% | - 0.090 (0.121) | 0.068 (0.002) | - 0.081 (0.532) |
| Carbohydrates (g/kg/d) | 0.082 (0.067) | - 0.144 (<0.001) | 0.084 (0.424) |
| Protein (g/kg/d) | - 0.003 (0.945) | - 0.077 (< 0.001) | - 0.046 (0.644) |
| Fat (g/kg/d) | 0.061 (0.218) | - 0.076 (< 0.001) | - 0.130 (0.249) |
| Cholesterol (mg/d) | - 0.167 (0.047) | 0.079 (0.010) | 0.033 (0.860) |
| SFA (g/d) | -0.942 (0.308) | 1.674 (<0.001) | -3.210 (0.122) |
| Trans fatty acids (g/d) | 0.081 (0.452) | 0.110 (0.016) | 0.500 (0.053) |
| MUFA (g/d) | - 0.010 (0.890) | 0.061 (0.026) | - 0.125 (0.421) |
| PUFA (g/d) | 0.050 (0.416) | 0.045 (0.073) | - 0.066 (0.636) |
| MUFA:PUFA:SFA balance | 0.071 (0.290) | - 0.055 (0.022) | - 0.014 (0.922) |
| Vitamins (only vitamins with at least one significant association are shown) | | | |
| B1 | - 0.022 (0.688) | 0.046 (0.023) | - 0.049 (0.686) |
| B2 | - 0.152 (0.005) | 0.077 (< 0.001) | - 0.187 (0.114) |
| B3 | - 0.002 (0.975) | 0.070 (0.003) | 0.061 (0.673) |
| B6 | - 0.169 (0.028) | 0.022 (0.433) | - 0.338 (0.057) |
| B12 | - 0.182 (0.032) | 0.075 (0.019) | - 0.042 (0.613) |
| D | - 0.371 (0.003) | 0.037 (0.420) | - 0.378 (0.207) |
| E | - 0.224 (0.008) | 0.057 (0.082) | - 0.245 (0.176) |
| Folate | - 0.043 (0.564) | 0.012 (0.652) | 0.339 (0.039) |
| Pantothenic acid | - 0.086 (0.782) | 0.053 (0.002) | - 0.030 (0.775) |
| Minerals (only minerals with et least one significant associations are shown) | | | |
| Ca | - 0.247 (< 0.001) | 0.087 (0.001) | - 0.250 (0.100) |
| P | - 0.116 (0.016) | 0.059 (0.001) | - 0.083 (0.425) |
| K | - 0.027 (0.551) | 0.039 (0.019) | - 0.036 (0.724) |
| Zn | - 0.047 (0.340) | 0.049 (0.006) | - 0.088 (0.409) |
| Na | - 0.133 (0.023) | 0.082 (< 0.001) | - 0.090 (0.502) |

E%, energy as percentage of total energy intake; IR%, intake as percentage of requirements; MUFA, Mono-Unsaturated Fatty Acids; PUFA, Poly-Unsaturated Fatty Acids; poor DQI-I, DQI-I below 60% of perfect; SFA, Saturated Fatty Acids.

Supplementary Table 7. Multiple regression analysis Model 2: Factors independently associated with the overall DQI-I and DQI-I domains, anergy and nutrient intake

| Dependent variables | Independent variables | | | |
| --- | --- | --- | --- | --- |
|  | Number of allergies | Age | Sex | Milk avoidance |
|  | B (p) | B (p) | B (p) | B (p) |
| Overall DQI-I | 0.009 (0.464) | - 0.007 (0.438) | 0.007 (0.873) | -0.058 (0.274) |
| DQI-I variety | - 0.015 (0.740) | 0.013 (0.542) | - 0.173 (0.079) | 0.037 (0.751) |
| DQI-I adequacy | - 0.018 (0.799) | - 0.010 (0.326) | 0.065 (0.384) | -0.008 (0.466) |
| DQI-I moderation | -0.049 (0.136) | - 0.025 (0.065) | 0.046 (0.510) | -0.201 (0.012) |
| DQI-I balance | - 0.186 (0.005) | - 0.017 (0.889) | 0.078 (0.636) | 0.469 (0.043) |
| Energy IR% | 5.039 (0.082) | - 4.614 (< 0.001) | - 0.206 (0.974) | 0.463 (0.949) |
| Carbohydrate E% | 0.766 (0.546) | 1.699 (< 0.001) | 4.00 (0.122) | - 2.773 (0.380) |
| Protein E% | - 0.992 (0.106) | 0.687 (0.008) | - 0.696 (0.607) | 0.762 (0.619) |
| Fat E% | 0.226 (0.818) | 1.013 (0.014) | - 3.303 (0.125) | 2.010 (0.410 |
| SFA E% (LOG_ | - 0.046 (0.404) | 0.024 (0.315) | 0.000 (0.997) | 0.495 (< 0.001) |
| Carbohydrates (g/kg/d) | 0.080 (0.082) | - 0.141 (<0.001) | 0.079 (0.459) | - 0.032 (0.784) |
| Protein (g/kg/d) | - 0.008 (0.868) | - 0.087 (< 0.001) | - 0.024 (0.810) | 0.117 (0.300) |
| Fat (g/kg/d) | 0.052 (0.295) | - 0.054 (0.017) | - 0.176 (0.098) | 0.065 (0.608) |
| Cholesterol (mg/d) | - 0.159 (0.064) | 0.071 (0.050) | 0.048 (0.798) | 0.093 (0.660) |
| SFA (g/d) | -0.017 (0.760) | 0.054 (0.034) | -0.101 (0.421) | 0.520 (< 0.001) |
| Trans (g/d) | 0.122 (0.318) | 0.082 (0.171) | 0.525 (0.043) | 0.252 (0.480) |
| MUFA (g/d) | - 0.018 (0.810) | 0.069 (0.040) | - 0.133 (0.397) | - 0.079 (0.674) |
| PUFA (g/d) | 0.024 (0.710) | 0.068 (0.027) | - 0.081 (0.560) | - 0219 (0.194) |
| MUFA:PUFA:SFA | - 0.024 (0.701) | -0.021 (0.429) | - 0.078 (0.541) | - 0.751 (< 0.001) |
| Vitamins (only vitamins with significant associations are shown) | | | | |
| B1 | - 0.016 (0.769) | 0.040 (0.076) | - 0.031 (0.803) | 0.094 (0.0.467) |
| B2 | - 0.130 (0.010) | 0.047 (0.020) | - 0.106 (0.361) | 0.397 (0.001) |
| B3 | - 0.005 (0.946) | 0.0730 (0.006) | 0.050 (0.376) | - 0.041 (0.797) |
| B6 | - 0.198 (0.010) | 0.075 (0.026) | - 0.410 (0.017) | - 0.553 (0.004) |
| B12 | - 0.158 (0.059) | 0.045 (0.193) | - 0.050 (0.777) | 0.372 (0.045) |
| D | - 0.384 (0.002) | 0.011 (0.830) | - 0.345 (0.255) | 0.372 (0.211) |
| E | - 0.257 (0.004) | 0.092 (0.034) | -0.290 (0.114) | - 0.295 (0.217) |
| Folate | - 0.038 (0.603) | - 0.003 (0.909) | 0.375 (0.022) | 0.274 (0.088) |
| Pantothenic acid | - 0.072 (0.140) | 0.037 (0.059) | - 0.006 (0.957) | 0.193 (0.100) |
| Minerals (only minerals with significant associations are shown) | | | | |
| Ca | - 0.210 (0.001) | 0.037 (0.164) | - 0.164 (0.248) | 0.596 (< 0.001) |
| P | - 0.093 (0.049) | 0.035 (0.067) | - 0.034 (0.736) | 0.288 (0.010) |
| Cu | 0.015 (0.064) | 0.016 (0.420) | 0.012 (0.010) | -0.94 (0.480) |
| K | - 0.013 (0.780) | 0.025 (0.191) | - 0.015 (0.882) | 0.155 (0.186) |
| Zn | - 0.23 (0.637) | 0.025 (0.212) | - 0.059 (0.564) | 0.290 (0.011) |
| Na | - 0.121 (0.040) | 0.067 (0.013) | - 0.056 (0.684) | 0.159 (0.290) |

E%, energy as percentage of total energy intake; IR%, intake as percentage of requirements; MUFA, Mono-Unsaturated Fatty Acids; PUFA, Poly-Unsaturated Fatty Acids; SFA, Saturated Fatty Acids.
